# *COL4A1* Mutations Cause Ocular Dysgenesis, Neuronal Localization Defects, and Myopathy in Mice and Walker-Warburg Syndrome in Humans

**DOI:** 10.1371/journal.pgen.1002062

**Published:** 2011-05-19

**Authors:** Cassandre Labelle-Dumais, David J. Dilworth, Emily P. Harrington, Michelle de Leau, David Lyons, Zhyldyz Kabaeva, M. Chiara Manzini, William B. Dobyns, Christopher A. Walsh, Daniel E. Michele, Douglas B. Gould

**Affiliations:** 1 Departments of Ophthalmology and Anatomy, Institute for Human Genetics, University of California San Francisco School of Medicine, San Francisco, California, United States of America; 2 Department of Molecular and Integrative Physiology, University of Michigan, Ann Arbor, Michigan, United States of America; 3 Division of Genetics and The Manton Center for Orphan Disease Research, Children's Hospital Boston, Howard Hughes Medical Institute, and Harvard Medical School, Boston, Massachusetts, United States of America; 4 Departments of Human Genetics, Neurology, and Pediatrics, University of Chicago, Chicago, Illinois, United States of America; University of Pennsylvania, United States of America

## Abstract

Muscle-eye-brain disease (MEB) and Walker Warburg Syndrome (WWS) belong to a spectrum of autosomal recessive diseases characterized by ocular dysgenesis, neuronal migration defects, and congenital muscular dystrophy. Until now, the pathophysiology of MEB/WWS has been attributed to alteration in dystroglycan post-translational modification. Here, we provide evidence that mutations in a gene coding for a major basement membrane protein, collagen IV alpha 1 (*COL4A1*), are a novel cause of MEB/WWS. Using a combination of histological, molecular, and biochemical approaches, we show that heterozygous *Col4a1* mutant mice have ocular dysgenesis, neuronal localization defects, and myopathy characteristic of MEB/WWS. Importantly, we identified putative heterozygous mutations in *COL4A1* in two MEB/WWS patients. Both mutations occur within conserved amino acids of the triple-helix-forming domain of the protein, and at least one mutation interferes with secretion of the mutant proteins, resulting instead in intracellular accumulation. Expression and posttranslational modification of dystroglycan is unaltered in *Col4a1* mutant mice indicating that *COL4A1* mutations represent a distinct pathogenic mechanism underlying MEB/WWS. These findings implicate a novel gene and a novel mechanism in the etiology of MEB/WWS and expand the clinical spectrum of *COL4A1*-associated disorders.

## Author Summary

Muscle-eye-brain disease (MEB) and Walker-Warburg Syndrome (WWS) are devastating childhood diseases that belong to a subgroup of congenital muscular dystrophies (CMDs) characterized by ocular dysgenesis, neuronal migration defects, and congenital myopathy. Genetic studies have revealed a number of genes involved in the etiology of CMDs, and subsequent studies show that alterations in dystroglycan glycosylation underlie MEB/WWS. However, over half of MEB/WWS patients do not have mutations in known genes encoding glycosyltransferases, suggesting that other genes are involved. Here, we describe a novel and genetically complex mouse model for MEB/WWS and identify putative heterozygous mutations in *COL4A1* in two MEB/WWS patients. We identify a novel gene implicated in the etiology of MEB/WWS, provide evidence of mechanistic heterogeneity for this subgroup of congenital muscular dystrophies, and develop an assay to test the functional significance of putative *COL4A1* mutations. Our findings represent the first evidence for a dominant mutation leading to MEB/WWS–like diseases and expand the spectrum of clinical disorders resulting from *Col4a1/COL4A1* mutations.

## Introduction

Congenital muscular dystrophies (CMDs) involving ocular and cerebral malformations are devastating childhood diseases. Fukuyama congenital muscular dystrophy, Muscle-Eye-Brain disease (MEB), and Walker-Warburg Syndrome (WWS) are clinically and mechanistically related forms of CMD [1]–[4]. Patients present at birth or as infants with muscle weakness, hypotonia or even severe myopathy leading to fatal respiratory insufficiency. Clinical presentation varies between patients but often includes myofiber necrosis and fibrosis, replacement by adipose tissue, split muscle fibers and the presence of non-peripheral nuclei. In addition, while most patients exhibit marked elevation of serum creatine kinase (CK), others have CK levels within the normal range [5].

Ocular and cerebral phenotypes demonstrate variable penetrance and expressivity between individual patients. For instance, ocular dysgenesis can occur in either, or both, the anterior (Peters' anomaly, Rieger syndrome, cataracts, buphthalmos and developmental glaucoma) and posterior portions of the eye (retinal dysplasia, retinal detachments and optic nerve hypoplasia) [6]–[8]. Variable neurological manifestations, including mental retardation and epilepsy, may be at least partially explained by cerebral cortical malformations including cobblestone lissencephaly, cerebellar hypoplasia, hydrocephalus and encephalocele.

Genetic and biochemical studies led to the identification of a number of genes involved in the etiology of CMDs and revealed that alterations in post-translational processing of dystroglycan underlie MEB/WWS [9]–[15]. In these ‘dystroglycanopathies’, hypoglycosylation of dystroglycan disrupts ligand binding and impairs muscle fiber attachment to the extracellular matrix. Central nervous system pathology is proposed to be secondary to defective interactions between radial glial cells and the pial basement membrane [16]–[18]. Despite these major advances, over half of MEB/WWS patients do not have mutations in known genes encoding glycosyltransferases [19], [20], suggesting that other genes in this pathway contribute to disease or that independent mechanisms are responsible. Identifying new pathways involved in MEB/WWS will be an important breakthrough that could open new avenues for understanding and ultimately treating CMDs.

We have recently discovered the first mutations in the gene coding for the ubiquitous basement membrane protein type IV collagen alpha 1 in mice (*Col4a1*) and humans (*COL4A1*) [21]. COL4A1 is the most abundant basement membrane protein and is ubiquitously present in basement membranes with few exceptions. The collagenous domain (a long stretch of Gly-Xaa-Yaa repeats that forms a triple helix) accounts for over 90% of the protein. Mutations in this triple helix-forming domain are well documented to be pathogenic in several types of collagens, including type IV collagens. The mutation we identified in mice (referred to as *Col4a1^Δex40^*) disrupts a splice acceptor site, causing exon 40 to be skipped and concomitant deletion of 17 amino acids from the collagen triple helical domain which interferes with proper folding, assembly and secretion of heterotrimeric COL4A1 and COL4A2 [21], [22]. To date, eleven out of twelve other *Col4a1* mutations reported in mice [23], [24] and seventeen out of twenty one *COL4A1* mutations identified in humans [21], [22], [25]–[34] are missense mutations within the triple helical domain, demonstrating that alterations of this domain are highly pathogenic.

In mice and humans, semi-dominant *Col4a1*/*COL4A1* mutations are highly pleiotropic with variable expressivity. The tissue distribution and severity of pathology depends on genetic and environmental factors but commonly include cerebrovascular diseases, ocular and renal defects [21]–[24], [35]. Here, we broaden this spectrum to include MEB/WWS. We show that, depending on the genetic context; *Col4a1^+/Δex40^* mice recapitulate the pathophysiological hallmarks of MEB/WWS, including ocular anterior segment dysgenesis, optic nerve hypoplasia, cortical lamination defects and myopathy. In addition, we identify heterozygous mutations in the triple–helix-forming domain of COL4A1 in two MEB/WWS patients. Together, these findings support *COL4A1* mutations as a novel genetic cause of MEB/WWS. Importantly, *COL4A1* is not directly related to post-translational modification of dystroglycan. We show that the mechanism is independent of dystroglycan glycosylation and instead is probably due to decreased COL4A1 levels in basement membranes. *COL4A1* mutations are pleiotropic and these data describe another clinically distinct group of diseases that can result from alterations in a single gene.

## Results

### Mislocalization and increased apoptosis of retinal ganglion cells cause optic nerve hypoplasia

**Figure 1:**
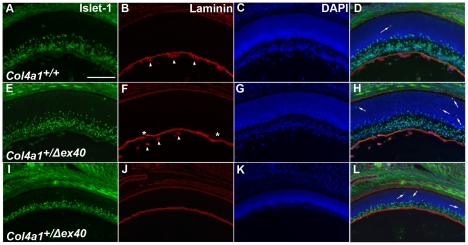
Mislocalization of retinal ganglion cells in *Col4a1^+/Δex40^* eyes. Representative position-matched transverse sections from *Col4a1^+/+^* (A–D) and *Col4a1^+/Δex40^* (E–L) eyes at E18 labeled with anti-ISL1 (green in A, E, and I), anti-laminin (red in B, F and J) and DAPI (blue in C, G, and K). Anti-ISL1 labeling revealed an increased number of RGCs in the outer retinas (arrows in D, H and L) of the mutant eyes. Labeling with anti-laminin antibody revealed focal disruptions in the inner limiting membrane in *Col4a1^+/Δex40^* mice (asterisks in F) and demonstrates that the retinal vasculature is adjacent to the inner limiting membrane in *Col4a1^+/+^* eyes but not in *Col4a1^+/Δex40^* eyes (arrowheads in B and F). Notably, in one extreme case, we detected no hyaloid vasculature and markedly reduced retinal thickness in the eye shown in I–L. Scale bar: 50 µm.

Ocular hallmarks of MEB/WWS include anterior segment dysgenesis and optic nerve hypoplasia. Depending on the genetic context, mutations in *Col4a1* can cause both anterior segment dysgenesis and optic nerve hypoplasia although the underlying pathogenic mechanism(s) remain unexplored [23], [24], [35]. During development, COL4A1 is present in the inner limiting membrane of the retina [36] and inner limiting membrane disruptions can perturb retinal ganglion cell (RGC) localization and cause apoptotic death [37]. To determine if excess RGC death during development caused optic nerve hypoplasia, developing retinas were immunolabeled for Islet-1 (ISL1) and Laminin, which mark newly specified RGCs [38] and basement membranes, respectively. At embryonic days (E) 14, E16 and E18, RGCs were located in the innermost part of the retina in *Col4a1^+/+^* mice, with only occasional ISL1 immunoreactivity detected in the outer retina (Figure 1D). In contrast, in *Col4a1^+/Δex40^* mice, the thickness of the ISL1 positive layer was highly variable and there were more displaced ISL1 positive cells detected in the outer retina (arrows in Figure 1H and 1L). Laminin immunoreactivity revealed focal disruptions in the inner limiting membranes of *Col4a1^+/Δex40^* animals (asterisks in Figure 1F) that were not observed in *Col4a1^+/+^* animals. Moreover, in contrast to *Col4a1^+/+^* eyes where the vasculature is closely associated with the inner limiting membrane, the hyaloid vasculature in *Col4a1^+/Δex40^* eyes is most often found in the vitreous (Figure 1B and 1F) and, in one extreme case, the posterior chamber was devoid of detectable vasculature (Figure 1J).

**Figure 2:**
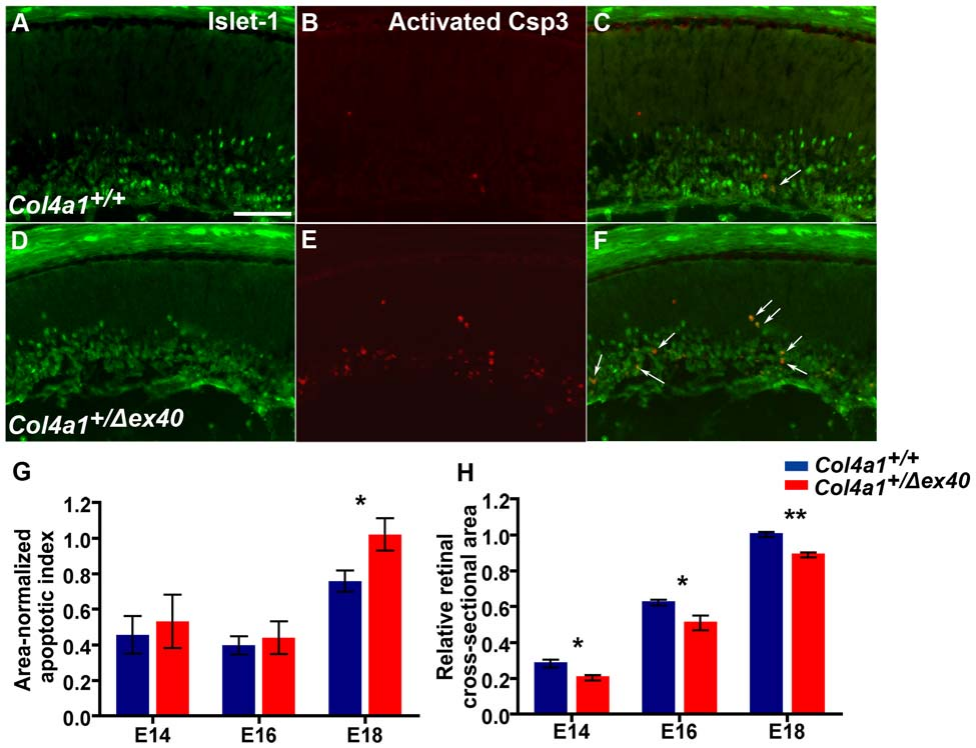
Increased apoptosis of Islet-1 positive retinal ganglion cells in *Col4a1^+/Δex40^* eyes. Representative eye sections from E18 *Col4a1^+/+^* mice (A–C) and *Col4a1^+/Δex40^* mice (D–F) co-labeled with anti-ISL1 (green in A and D) and anti-activated Caspase-3 (red in B and E). Co-labeling for ISL1 and Caspase-3 revealed an increase in the number of apoptotic retinal ganglion cells in *Col4a1^+/Δex40^* eyes. *Col4a1^+/Δex40^* eyes were significantly smaller than *Col4a1^+/+^* eyes at all ages examined (H; E14 p = 0.01; E16 p = 0.04; E18 p<10*^−5^* comparing at least 6 eyes for *Col4a1^+/+^* (blue) and *Col4a1^+/Δex40^* (red) at each age). After correcting for size, there was a significant increase in the number of apoptotic retinal ganglion cells in *Col4a1^+/Δex40^* eyes at E18 (G; p = 0.02 comparing at least 6 eyes for *Col4a1^+/+^* (blue) and *Col4a1^+/Δex40^* (red) at each age). In G and H, data are presented as mean +/− SEM. Scale bar: 50 µm.

During normal retinal development, approximately 50% of RGCs undergo programmed cell death as the visual system matures [39], [40]. Induced or genetic disruption of the inner limiting membrane perturbs RGC localization and leads to RGC apoptosis during embryogenesis [37]. Therefore, we hypothesized that *Col4a1^+/Δex40^* mice might exhibit increased RCG apoptosis. To test this, we co-labeled retinal sections with antibodies against ISL1 and activated Caspase-3 (Figure 2A–2F) and calculated the apoptotic index by counting the number of ISL1/Caspase 3 double-labeled cells (Figure 2G). As retinas from *Col4a1^+/Δex40^* mice were smaller than retinas from *Col4a1^+/+^* mice (Figure 2H), we normalized the number of double-labeled cells to the retinal cross-sectional area. *Col4a1^+/+^* mice had low levels of ganglion cell apoptosis at E14 and E16 that increased approximately 2-fold by E18. Although apoptotic rates in *Col4a1^+/Δex40^* mice at E14 and E16 were not significantly different from those observed in *Col4a1^+/+^* mice, there was a significant increase in apoptosis of ISL1 positive cells in *Col4a1^+/Δex40^* eyes compared to *Col4a1^+/+^* eyes at E18 (p<0.05) – especially among mislocalized ISL1 labeled cells (Figure 2F). Of note, one E18 *Col4a1^+/Δex40^* eye had an extremely high apoptotic index (12.9) that was considered an outlier and was removed from subsequent statistical analyses (see Materials and Methods). Interestingly, we did not observe vasculature in the posterior chamber of this eye suggesting that retinal vasculature might directly contribute to the inner limiting membrane or affect ganglion cell viability in other ways. Together, our data support that *Col4a1* mutation leads to focal disruptions of the inner limiting membrane and that optic nerve hypoplasia results both from reduced production of retinal neurons and from mislocalization and subsequent apoptosis of ganglion cells during development.

### *Col4a1^+/Δex40^* mice display cerebral cortical malformations and cerebral neuronal localization defects characteristic of cobblestone lissencephaly

**Figure 3:**
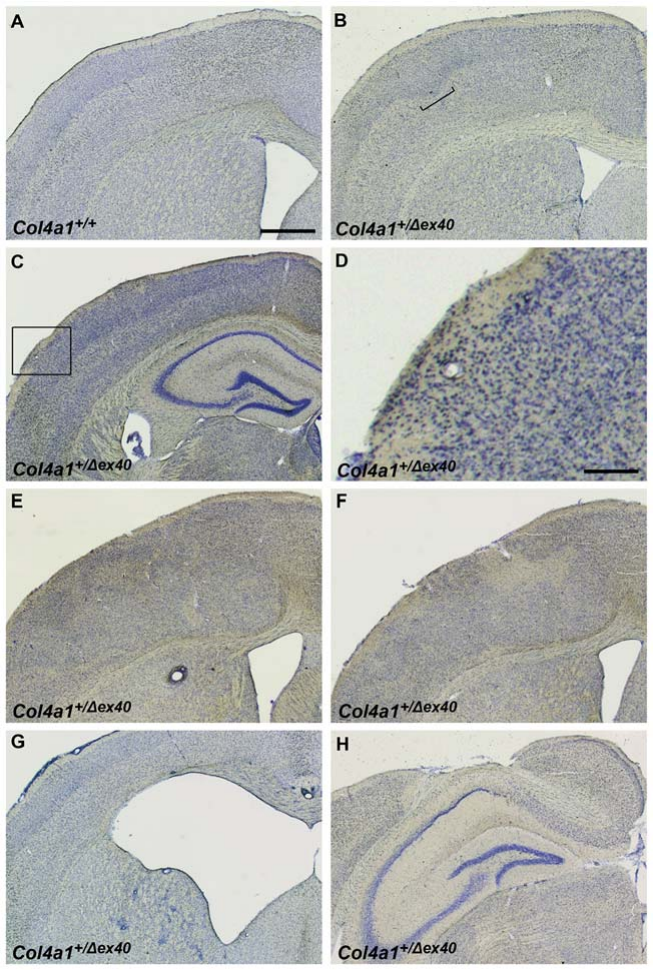
*Col4a1^+/Δex40^* mice have focal and variable cortical malformations. Representative cresyl violet stained coronal brain sections from adult *Col4a1^+/+^* (A) and *Col4a1^+/Δex40^* (B–H) revealed cortical malformations characteristic of cobblestone lissencephaly in all mutant brains examined (n = 6). In contrast to the well-defined cortical lamination observed in *Col4a1^+/+^* brains, *Col4a1^+/Δex40^* brains had variable abnormalities including disorganized cortical lamination (bracket in B), ectopias (C, enlarged in D), heterotopic regions (E and F), enlarged ventricles (G) and, occasionally, severe cortical malformations (H). Scale bars: A–C and E–H, 800 µm; d, 200 µm.

**Figure 4:**
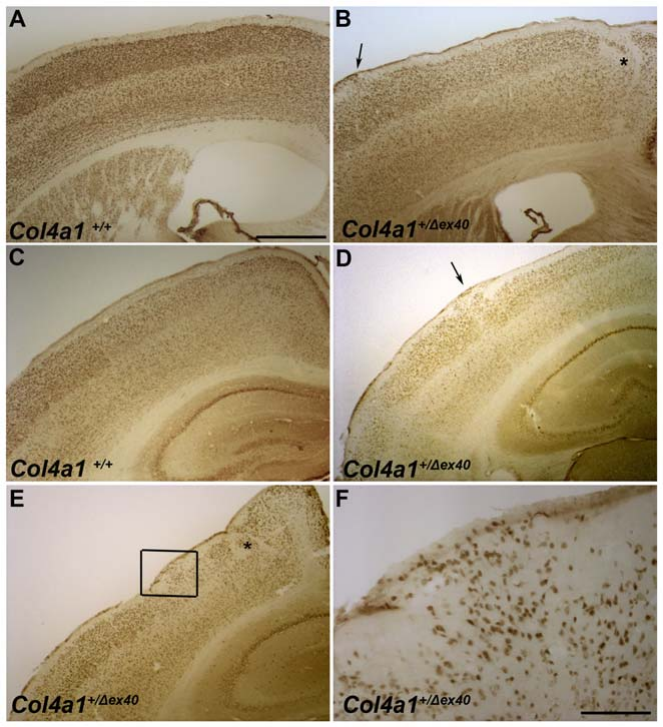
*Col4a1^+/Δex40^* mice display cortical neuronal localization defects. Representative images of NeuN-labeled coronal brain sections from adult *Col4a1^+/+^* (A and C) and *Col4a1^+/Δex40^* (B and D–F) mice revealed heterotopic regions (asterisks in B and E) and ectopias (arrows in B and D) in all mutant brains examined. NeuN labeling of ectopic cells located in the marginal zone (F, enlarged from box in E) confirmed their neuronal identity. Scale bars: A–E, 500 µm; F, 125 µm.

Based on our observations in the retina, we predicted that *Col4a1^+/Δex40^* mice might also show pial basement membrane disruptions and cerebral cortical lamination defects that model cobblestone lissencephaly seen in MEB/WWS. To test our hypothesis, we stained coronal brain sections from adult *Col4a1^+/+^* and *Col4a1^+/Δex40^* mice with cresyl violet and detected abnormalities in all of the *Col4a1^+/Δex40^* but none of the *Col4a1^+/+^* mice examined (Figure 3). All *Col4a1^+/Δex40^* mice had focal and variable cerebral cortex lamination defects ranging from mild distortions and ectopias to severe heterotopic regions devoid of obvious lamination (Figure 3B–3F). Occasionally, *Col4a1^+/Δex40^* mice displayed enlarged ventricles or major structural abnormalities (Figure 3G and 3H). Immunolabeling with the pan–neuronal marker NeuN confirmed the neuronal identity of the ectopic cells (Figure 4). *Col4a1^+/Δex40^* mice also had subtle but consistent defects within the hippocampus (Figure S1). The CA1, CA3 and dentate gyrus layers of *Col4a1^+/Δex40^* mice were less tightly organized and generally more dispersed compared to *Col4a1^+/+^* mice and local perturbations were common. As it is the case in other animal models of MEB/WWS [41], [42], enhanced glial fibrillary acid protein (GFAP) immunoreactivity, which is reflective of astrocytic gliosis, was observed in the hippocampus and cerebral cortex of *Col4a1^+/Δex40^* mice (Figure S2).

### Neuronal localization defects in *Col4a1^+/Δex40^* mice are congenital

**Figure 5:**
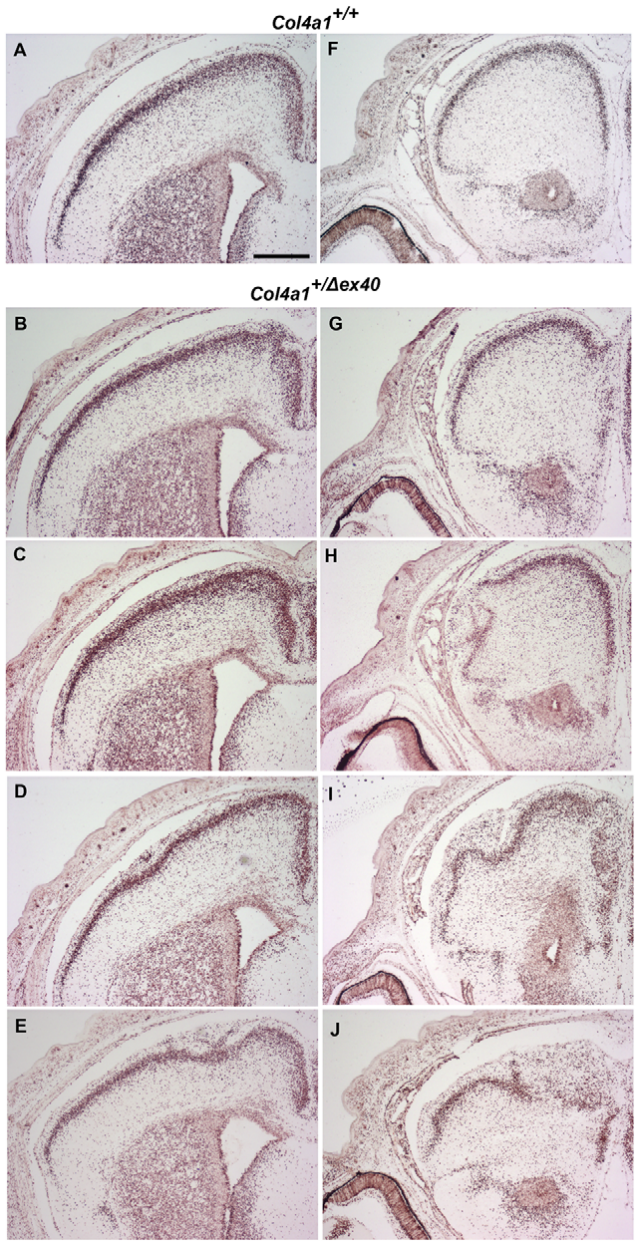
Focal and variable cortical neuronal localization defects are developmental. Embryos pulsed-labeled with BrdU at E14 were harvested at birth and BrdU was immunolabeled in position-matched coronal brain sections of *Col4a1^+/+^* (A and F) and *Col4a1^+/Δex40^* mice (B–E and G–J). In contrast to *Col4a1^+/+^* controls, in which BrdU-labeled cells are distributed in a uniform layer in the outer cortex, all *Col4a1^+/Δex40^* brains had focal neuronal localization defects that ranged in severity from diffuse cell layers to ectopias and severely disorganized cortical lamination (note gradient of severity from B through E and from G through J). Similar results were observed in embryos labeled at E16. Scale bar: 500 µm.

**Figure 6:**
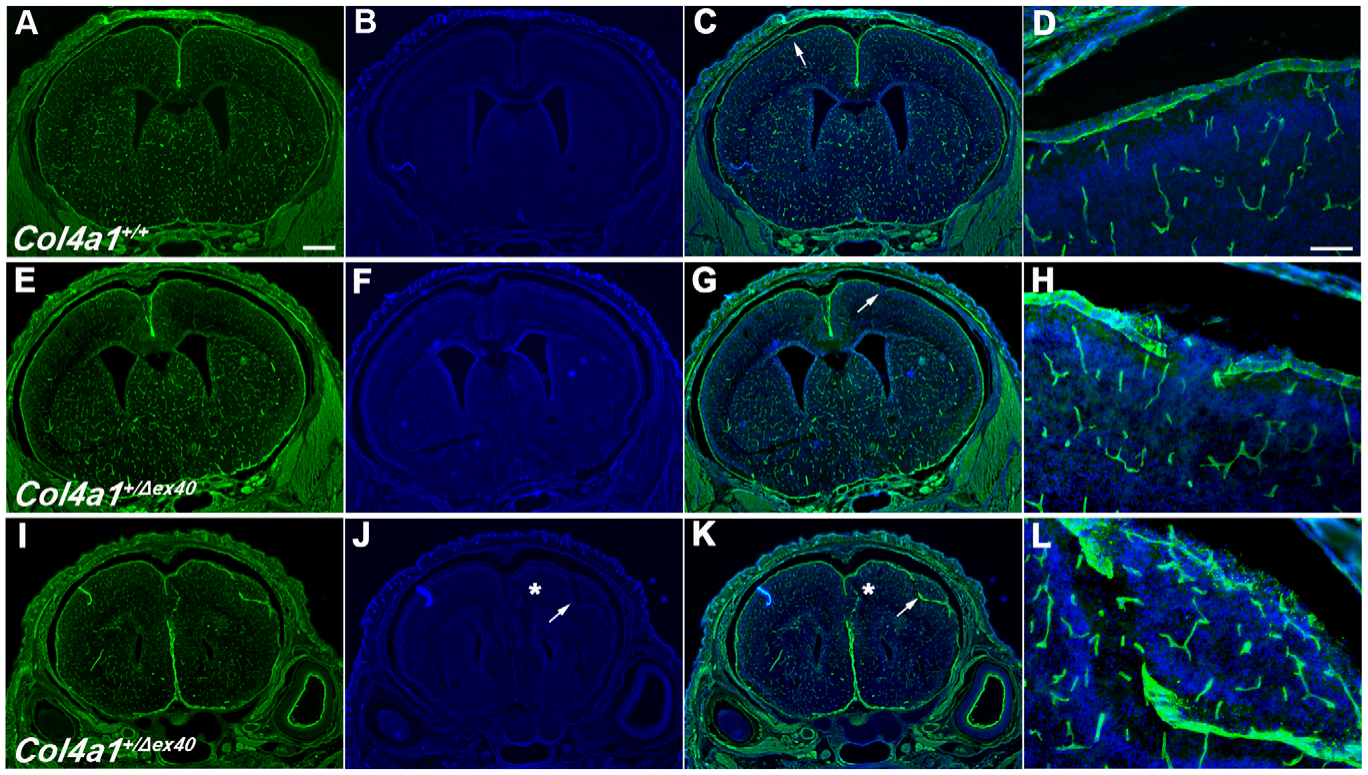
*Col4a1^+/Δex40^* mice have focal breaches of the pial basement membrane during development. Representative images of laminin labeled (green) coronal brain sections from P0 *Col4a1^+/+^* (A–D) and *Col4a1^+/Δex40^* (E–L) revealed breaches of the pial basement membrane in mutant animals. All sections of *Col4a1^+/+^* brains examined (A–D) showed intact pial membranes (higher magnifications in D). In contrast, position-matched *Col4a1^+/Δex40^* brains display multiple focal disruptions of the pial basement membrane (arrows and asterisks in G and K) that are shown in higher magnification in H, and L. Notably, the high magnification image in L shows ectopic cells (DAPI in blue) that have breached the pial membrane adjacent to a disruption. Scale bars: A–C, E–G, and I–K, 500 µm; D, H and L, 50 µm.

To determine if cortical malformations were congenital or acquired, we used bromodeoxyuridine (BrdU) pulse labeling *in utero* to evaluate the localization of neurons that underwent terminal cell division during defined stages of embryogenesis. The locations of cells labeled at E14 or E16 were determined at birth (P0). In all *Col4a1^+/+^* mice, BrdU-labeled neurons were uniformly distributed primarily in the superficial cortex (Figure 5A and 5F). In contrast, the distribution of BrdU-labeled cells in mutant animals demonstrated that cortical lamination was disorganized. Focal and variable lamination defects were completely penetrant in *Col4a1^+/Δex40^* mice (Figure 5B–5E and 5G–5J). Laminin labeling of basement membranes in P0 mice revealed discontinuous pial basement membranes in all mutant mice, notably in areas adjacent to ectopias (Figure 6). Together, these findings demonstrate that *Col4a1^+/Δex40^* mice have abnormal neuronal localization typical of cobblestone lissencephaly observed in MEB/WWS patients and suggest that these congenital defects are secondary to breaches in the pial basement membrane.

### Genetically modifiable myopathy in *Col4a1^+/Δex40^* mice

**Figure 7:**
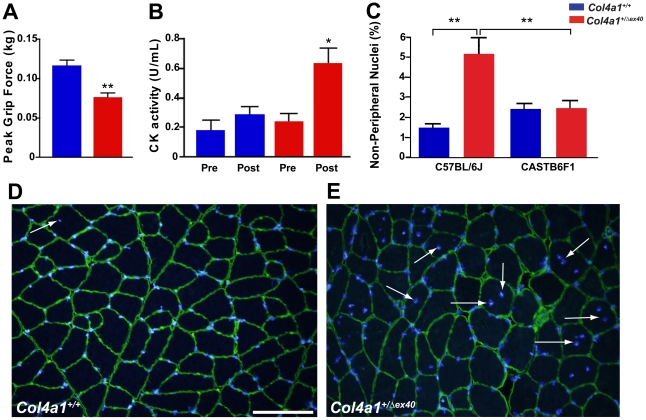
*Col4a1^+/Δex40^* mice have functional, biochemical, histological, and genetically–complex myopathy. (A) Comparison of the peak grip force (mean +/−SEM) between *Col4a1^+/+^* (blue; n = 7) and *Col4a1^+/Δex40^* (red; n = 7) mice at 3 months of age revealed a significant reduction in *Col4a1^+/Δex40^* mice (** indicates p<0.01). (B) Serum creatine kinase (CK) activity was compared (mean +/−SEM) between *Col4a1^+/+^* (blue; n = 7) and *Col4a1^+/Δex40^* (red; n = 7) mice before (Pre) and after (Post) exercise (* indicates p<0.05 vs all other groups). (C–E) Comparison of non-peripheral nuclei (arrows in D and E; mean +/−SEM in C) between *Col4a1^+/+^* (counting of 17830 fibers in 5 muscles from 4 mice) and *Col4a1^+/Δex40^* (counting of 13820 fibers in 4 muscles from 4 mice) mice (14 to 16 months old) revealed a significant increase in the number of non-peripheral nuclei in *Col4a1^+/Δex40^* mice on the C57BL/6J background (control mean = 1.5%; mutant mean = 5.2%; p<0.0005). This difference is modified by the genetic context. There was no difference between *Col4a1^+/+^* and *Col4a1^+/Δex40^* CASTB6F1 mice and there was a significant rescue of non-peripheral nuclei in CAST/B6F1 *Col4a1^+/Δex40^* mice compared to C57BL/6J *Col4a1^+/Δex40^* mice (p<0.005). Scale bar: 100 µm.

Because *Col4a1^+/ex40^* mice display ocular and cerebral abnormalities characteristic of MEB/WWS, we hypothesized that they would also have myopathy. To test this, we first confirmed that COL4A1 was present in skeletal muscle basement membrane by immunolabeling (Figure S3) and performed functional, biochemical and histological analyses of muscles from young and aged *Col4a1^+/+^* and *Col4a1^+/Δex40^* mice. At 3 months of age, *Col4a1^+/Δex40^* mice performed significantly worse than controls in a test of peak grip force (Figure 7A). Next, we analyzed serum CK activity before and after exercise. We found no significant difference in CK levels between control and mutant mice at baseline, however, *Col4a1^+/Δex40^* mice had a significant elevation in CK activity following exercise compared to pre-exercise *Col4a1^+/Δex40^* and post-exercise *Col4a1^+/+^* mice (Figure 7B). Consistent with these functional and biochemical data, we also detected histological differences between *Col4a1^+/+^* and *Col4a1^+/Δex40^* muscles. Compared to *Col4a1^+/+^* littermates, *Col4a1^+/Δex40^* mice had occasional split muscle fibers and a significant increase in the number of non-peripheral nuclei – a measure of myopathy (Figure 7C–7E). Importantly, the severity of myopathy was not markedly affected by age.

We have shown previously that ocular dysgenesis is genetic context–dependent and that mutant F1 progeny of C57BL/6J and CAST/EiJ crosses (CASTB6F1) are morphologically rescued [35]. As shown in Figure 7C, in contrast to what is observed on the C57BL/6J background, the number of non-peripheral nuclei was not increased in CASTB6F1 *Col4a1^+/Δex40^* mice compared to their *Col4a1^+/+^* littermates. Moreover, there were significantly fewer muscle fibers with non-peripheral nuclei in CASTB6F1 *Col4a1^+/Δex40^* mice compared to C57BL/6J *Col4a1^+/Δex40^* mice (p<0.05), indicating that the CAST/EiJ strain has one or more loci that can also ameliorate *Col4a1*-induced myopathy.

### Identification of *COL4A1* mutations in human MEB/WWS patients

*Col4a1^+/Δex40^* mice display multiple hallmarks of MEB/WWS raising the possibility that *COL4A1* mutations might cause CMD-like diseases in human patients. To test this, we performed direct sequence analysis of genomic DNA from a cohort of 27 patients with CMD (see Table S1 for primers). Fifteen patients had diagnoses of WWS, two had diagnoses of MEB, and nine were not specifically classified as either but had CMD with variable ocular and cerebral involvement. To enrich for patients that may have dominant or semi-dominant mutations, rather than recessive mutations, most of the patients (23 out of 27) were chosen from non-consanguineous families. Finally, all patients were negative for mutations in genes currently known to underlie MEB/WWS-like diseases including *LARGE*, *POMT1*, *POMT2*, *POMGNT1*, *FKTN* and *FKRP*.

**Figure 8:**
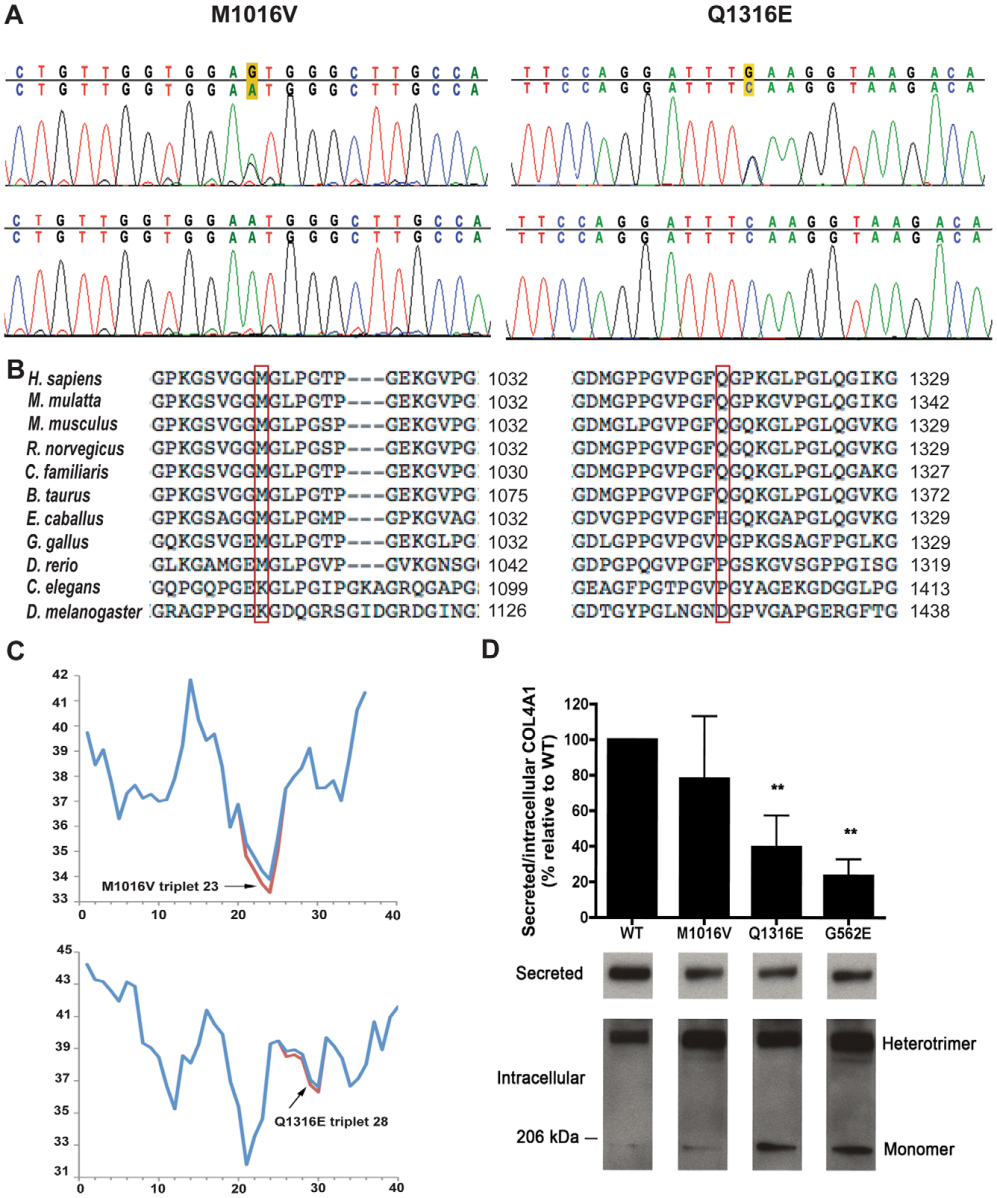
Identification of *COL4A1* missense mutations in two human patients. Direct sequence analysis of patient DNA revealed two heterozygous missense mutations (A, top panels) that were not found in control patients (A, bottom panels). (B) Protein sequence alignments with multiple species indicate a very strong degree of conservation of the altered amino acid for both mutations. Methionine at position 1016 is conserved in all species tested except for *C. elegans* and *Drosophila* where this amino acid is a lysine. Glutamine at position 1316 is also highly conserved and is never a glutamate residue. (C) Triple helix thermal stability was calculated by amino acid sequence and is plotted for the length of the COL4A1 protein. Arrows indicate the positions for each mutation and the effects of the mutations on calculated thermal stability are indicated in red. (D) For quantitative analysis of the ratio of secreted to intracellular mutant COL4A1 protein levels, values are expressed as percentage of the wild-type COL4A1 ratio and are presented as mean +/− SEM. Representative Western blot images for secreted and intracellular COL4A1 are shown below the graph. Nine independent Western blot experiments were performed using 6 independent clones per mutation for this analysis, **P<0.01.

We identified several coding and non-coding sequence variants in *COL4A1* (Tables S2, S3, S4). Twelve coding variants were silent and were either observed in controls or were not predicted to be splice–site-altering variants [43]. We identified four non-synonymous coding variants. Two of the four non-synonymous variants were previously identified SNPs that were highly polymorphic in patients and in controls and therefore deemed unlikely to be pathogenic. The two remaining SNPs were rare, missense variants that have not been previously reported in dbSNP and were heterozygous in independent patients. The first mutation was identified in a patient diagnosed with WWS that had lissencephaly, hydrocephalus, Dandy-Walker malformation, optic nerve hypoplasia and was hypotonic with CMD (see Figure S4 and Text S1 for clinical details). An adenine to guanine transition (A3046G) in exon 36 substituted a methionine residue for a valine residue within the triple helical domain at amino acid number 1016 (p.M1016V) and was not detected in 282 control chromosomes (Figure 8A).

The second mutation was identified in a patient with unspecified CMD with ocular and cerebral involvement. The patient had mild gyral abnormalities, hydrocephalus, retinal dysplasia, seizures and elevated CK (see Figure S4 and Text S1 for clinical details). A cytosine to guanine transversion (C3946G) in exon 44 substituted a glutamine residue for a glutamate residue within the triple helical domain of the protein at amino acid number 1316 (p.Q1316E) (Figure 8A). Although this cytosine to guanine transversion was not detected in 286 control chromosomes, it was present in the paternal DNA.

### Functional analysis of putative *COL4A1* mutations

The first variant, *COL4A1^M1016V^*, is a mutation of a methionine residue in the Y position of the Gly-Xaa-Yaa repeat in the triple helix-forming domain. This amino acid is highly conserved and the analogous residue is a methionine in all vertebrate orthologues analyzed (Figure 8B) and in the COL4A1 paralogues COL4A3 and COL4A5 (data not shown). Moreover, there is precedence for a Y position methionine to valine mutation in *COL4A5* causing Alport syndrome [44]. The second variant, *COL4A1^Q1316E^*, is a mutation of a glutamine in the Y position residue within the triple helix-forming domain. This glutamine residue is conserved in most vertebrates (Figure 8B). There is a strong preference for basic residues in the Y position of the Gly-Xaa-Yaa repeat and the mutation represents a substitution from a neutral amino acid to an acidic amino acid. Pathogenic mutations within the collagenous domain often perturb triple helix assembly and mutations in regions of low thermal stability are predicted to be more disruptive [45]. According to an algorithm that predicts collagen stability [46], both mutations modestly reduce the thermal stability of their respective region (Figure 8C) although the biological relevance of this is equivocal.

Pathogenic mutations in the triple helix forming domain of several types of collagens impair secretion of the collagen heterotrimers and concomitantly, misfolded proteins accumulate within cells. To assess the functional significance of the *COL4A1^M1016V^* and *COL4A1^Q1316E^* mutations, we developed an assay to test the impact of the mutant proteins in a human cell line. We stably transfected HT1080 cells with wild–type or mutant *COL4A1* cDNAs and determined the relative levels of intracellular and secreted COL4A1. To validate the assay, we tested the effect of the *COL4A1^G562E^* mutant allele that is established to cause familial small vessel disease in human patients [22], [26]. As we predicted, when compared to HT1080 cells transfected with wild–type *COL4A1*, significant intracellular accumulation of COL4A1 and concurrent decrease in secreted COL4A1 was observed in cells transfected with the *COL4A1^G562E^* mutant cDNA (Figure 8D). The two putative pathogenic mutations were functionally tested using the same assay. Overall, the ratio of secreted/intracellular COL4A1 for the *COL4A1^M1016V^* mutation was reduced, however the results were variable (n = 9) and did not reach statistical significance. In contrast, and similar to the established *COL4A1^G562E^* mutation, the *COL4A1^Q1316E^* mutation clearly impaired COL4A1 secretion leading to intracellular accumulation (p<0.001), supporting the hypothesis that the *COL4A1^Q1316E^* mutation is pathogenic. Importantly, the accumulation of both the monomeric and heterotrimeric forms of COL4A1 suggests that the *COL4A1^G562E^* and *COL4A1^Q1316E^* mutations impaired triple helix assembly and/or stability.

### The mechanism of *Col4a1*-induced disease is independent of dystroglycan glycosylation

**Figure 9:**
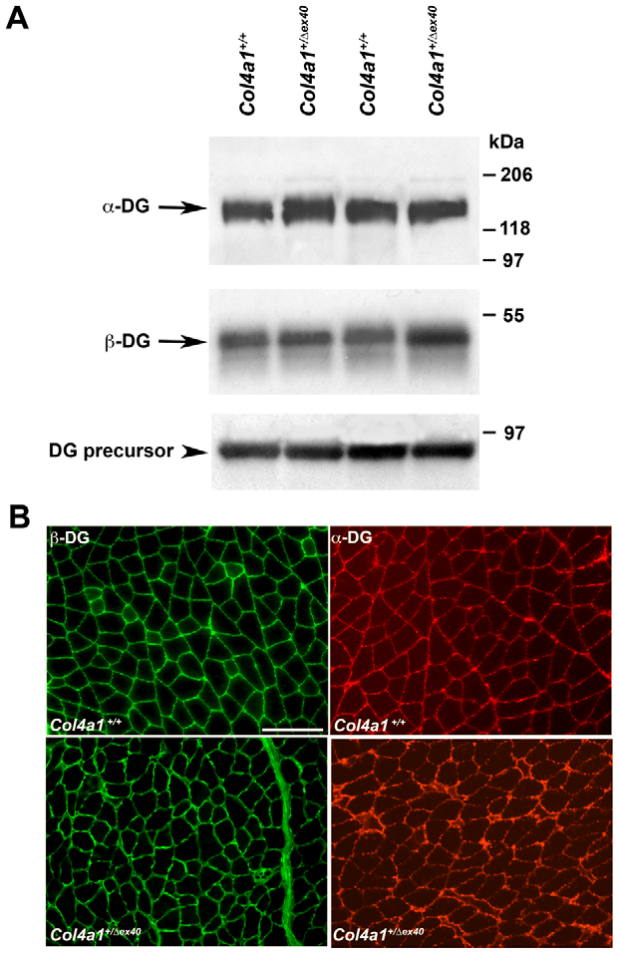
The pathogenic mechanism by which *Col4a1* mutation causes MEB/WWS phenotypes is independent of dystroglycan glycosylation. (A) Western blot analysis of total protein lysates from quadriceps biopsy using antibody for dystroglycan precursor protein (bottom) did not reveal a difference in dystroglycan precursor expression between *Col4a1^+/+^* and *Col4a1^+/Δex40^* mice. To test for differences in quantity or mobility (glycosylation) of α-dystroglycan and β-dystroglycan, lysates were enriched for glycosylated proteins by WGA binding. WGA-enriched fraction of quadriceps protein extracts did not reveal differences in quantity or glycosylation of either α-dystroglycan (top) or β-dystroglycan (middle) between *Col4a1^+/+^* and *Col4a1^+/Δex40^* mice. (B) Immunohistochemical labeling of muscle sections with antibodies against the glycosylated form of α–dystroglycan (red) and β-dystroglycan (green) show similar patterns of α– and β-dystroglycan expression between *Col4a1^+/+^*and *Col4a1^+/Δex40^* mice.

Biochemical analyses revealed that known MEB/WWS-causing mutations act via hypoglycosylation of dystroglycan [11]. Although COL4A1 is not directly involved in post-translational dystroglycan modification, misfolded COL4A1^Δex40^ proteins in the endoplasmic reticulum (ER) might indirectly impair dystroglycan post-translational modification. Similarly, ER stress can produce reactive oxygen species [47]–[50] and exposure to reactive oxygen species can result in dystroglycan de–glycosylation [51]. Thus, *Col4a1*-induced pathogenesis might still act indirectly via dystroglycan hypoglycosylation or de–glycosylation. To test for alterations in dystroglycan expression and/or post-translational processing, we performed Western blot analysis on wheat germ agglutinin (WGA)-enriched skeletal muscle extracts and compared the amount and mobility of α– and β– dystroglycan between *Col4a1^+/+^* and *Col4a1^+/Δex40^* mice using a polyclonal β–dystroglycan antibody and the IIH6 α–dystroglycan antibody that recognizes the fully glycosylated, functional form of α–dystroglycan [11]. Immunoreactivity to, and mobility of, the precursor dystroglycan protein (WGA-unbound fraction) and α– and β– dystroglycan (WGA-bound, glycoprotein enriched fraction) were indistinguishable between *Col4a1^+/+^* and *Col4a1^+/Δex40^* mice (Figure 9). Consistent with this finding, immunolabeling of muscle sections for α– and β– dystroglycan showed similar expression patterns in the sarcolemma membrane of *Col4a1^+/+^* and *Col4a1^+/Δex40^* mice. These data indicate that dystroglycan expression, localization and post-translational modification are not altered in *Col4a1^+/Δex40^* mice and that myopathy arises via disruption of the basal lamina.

## Discussion

In this study, we show that *COL4A1* mutations cause multiple pathophysiological hallmarks of MEB/WWS in mice and possibly MEB/WWS in human patients. Mice harboring a semi-dominant *Col4a1* mutation have ocular dysgenesis, cerebral cortical lamination defects and myopathy. Optic nerve hypoplasia results, at least in part, from mislocalization and increased apoptosis of RGCs during development. Cerebral cortical malformations range from subtle lamination defects to large structural abnormalities. Myopathy is mild, but consistent and significant, and is exacerbated by exercise but not by age. Importantly, myopathy was genetic background-dependent, which implies that in resistant genetic contexts *Col4a1*- induced myopathy might not be detected but that on permissive genetic backgrounds, myopathy could be severe. Two out of 27 MEB/WWS patients tested had *COL4A1* mutations and in at least one case the mutation interfered with protein secretion. Moreover, unlike all other known causes of MEB/WWS, the cellular mechanism underlying *Col4a1*-induced pathology is independent of dystroglycan post-translational modification.

Until now, the precise pathogenic mechanism resulting from *COL4A1* mutations in human patients was poorly characterized and was only presumed to impair protein secretion and cause intracellular accumulation. Here, we have established and validated a cellular assay that demonstrates this effect. However, the relative contribution of COL4A1 deficiency in the basement membrane versus toxic intracellular accumulation to pathogenesis is difficult to dissociate and other possible mechanisms exist. Although the ratio of secreted to intracellular protein for the *COL4A1^M1016V^* mutation was not statistically different from wild–type there was an overall reduction in the ratio. It is important to bear in mind that absence of statistical significance does not necessarily imply absence of biological relevance. For instance, more efficient intracellular degradation of some misfolded proteins could prevent detection of an altered ratio of secreted to intracellular COL4A1 for some mutations. Alternatively, these data could indicate a third pathogenic mechanism whereby mutant proteins are secreted and exert a detrimental extracellular effect. In support of such a mechanism, the *COL4A1^M1016V^* mutation occurs within a putative binding site for the matricellular glycoprotein SPARC (secreted protein acid and rich in cysteine) [52] and could therefore act by disrupting protein-protein interaction in the extracellular matrix.

These variants represent the first heterozygous mutations in MEB/WWS patients. Although generally considered to be recessive, the inheritance pattern in many MEB/WWS patients is unknown. Phenotypic variability, reduced penetrance, and *de novo* mutations can all explain how dominant mutations might appear recessive in small families. Here, we observed non–penetrance of the *COL4A1^Q1316E^* mutation in the father of the affected child. Families with *COL4A1* mutations are only now starting to be identified but apparent non-penetrance, and asymptomatic carriers have already been reported [29], [53]. Importantly, *Col4a1-*associated phenotypes in mice are not only influenced by environmental factors but are also genetic context–dependent, and reduced penetrance could reflect modifier loci. Alternatively, genetic mosaicism of dominant collagen mutations can explain asymptomatic carriers [54].

We identified mutations in two out of 27 CMD patients tested. DNA was limiting and prevented us from also sequencing *COL4A2*; however, evidence from mice and *C. elegans* support that mutations in *COL4A2* may cause phenotypes similar to those resulting from mutations in *COL4A1* [24], [45], [55]. It will be very important in the future to determine whether the *COL4A1* findings extend to *COL4A2*. This could have an even broader relevance as *COL4A1* mutations are pleiotropic with variable penetrance and expressivity from organ to organ. It is possible that *COL4A1* mutations underlie CMD in patients with different, non-MEB/WWS-like, subclasses of the disease. Based on our current data, the most likely patients are those with mild myopathy and those with clinical manifestations that overlap with some of the other *COL4A1*-related phenotypes described in non-MEB/WWS syndromes including porencephaly, renal disease and cerebrovascular disease. Collectively, these findings provide the impetus for *COL4A1* and *COL4A2* mutation analysis in further cohorts of patients with CMD and/or congenital cerebral malformations.

*COL4A1* mutations have been identified in families with a spectrum of diseases affecting the cerebral vasculature, although pathologies of the eyes, kidneys and muscles have also been reported [21], [22], [25]–[29], [31], [33], [56], [57]. Notably, *COL4A1* appears to be an important genetic cause of porencephaly – a condition usually diagnosed in infants and characterized by large cerebral cystic cavities that communicate with the ventricles. Importantly, the cortical malformations present in MEB/WWS and described in the current manuscript are clinically and mechanistically distinct from porencephalic cavities, which are predicted to result from pre– or peri–natal hemorrhages in the germinal matrix. *Col4a1* mutations are pleiotropic and our findings expand the phenotypic spectrum resulting from *Col4a1* mutations in mice. We propose that *COL4A1* mutations in human patients will reflect the pleiotropy observed in mice and will be involved in the pathogenesis of diseases clinically distinct from those reported previously. Optic nerve hypoplasia and cortical neuronal migration defects have not been described in human patients with *COL4A1* mutations and a role for *COL4A1* or *COL4A2* in MEB/WWS-like diseases was not previously suspected. Interestingly, six families with *COL4A1* mutations and cerebrovascular disease also had muscle cramps and CK elevation [28], [33]. Although the degree of CK elevation in *Col4a1* mutant mice is less than that observed in dystrophin-glycoprotein complex related disorders, it is comparable to that seen in other types of dystrophies involving extracellular matrix proteins, including collagen VI -associated Bethlem myopathy and Ullrich CMD. While the exact mechanism underlying myopathy in collagen VI-related disorders is unclear, several lines of evidence suggest that mitochondrial dysfunction may be an important player [58].

We show that genetic context is an important factor contributing to the variable penetrance and severity of *Col4a1*-related diseases and that the CAST/EiJ strain can modify myopathy. These findings also imply that certain genetic contexts might exacerbate myopathy and that *COL4A1* mutations could also cause severe CMD. Importantly, genetic modifiers can be general or tissue specific and tissue-specific modification could help explain how mutations in a single gene can contribute to such diverse phenotypes. We also demonstrate that gene–environment interactions contribute to phenotypic variability. We have previously reported that cesarean delivery can reduce the risk of perinatal intracerebral hemorrhages and here we show that elevation in CK activity is exercise–dependent. Allelic differences could also help explain variable expressivity and severity of *COL4A1*-associated diseases between patients. For instance, a mutation that affects heterotrimer assembly might have broader effects than a mutation that specifically affects interactions with cell surface receptors, growth factors or other extracellular matrix molecules. Interestingly allelic differences are already emerging pointing to genotype/phenotype correlations in some families [28], [33].

Pial basement membrane integrity is critical for normal cortical development and insufficient COL4A1 in basement membranes renders them prone to disruption. Breaches in the pial membrane cause alterations in cortical neuronal distribution [17], [59], [60]. These are not intrinsic neuronal migration defects but are secondary to disorganization of the cortical marginal zone, and to defects in anchorage of glial endfeet and formation of the Cajal-Retzius cell layer [16]. Reactive gliosis and increased GFAP labeling may reflect broader breaches of the glia limitans including the blood brain barrier [11], [42]. Thus, while some structural defects might be due to disruptions of the pial basement membrane, other pathology might be attributed to disruptions of vascular basement membranes and/or defects in blood brain barrier function.

Understanding the precise mechanism underlying myopathy is complicated by the presence of COL4A1 in basement membranes of the sarcolemma, myotendonous junctions and neuromuscular junctions. Notably, *Col4a1* mutant mice have transient neuromuscular junction abnormalities that reportedly resolve by 3 weeks of age [61]. Determination of the primary site of pathogenesis for myopathy will likely require conditional expression of the *Col4a1* mutation. However, evidence from other model systems suggests that the primary mechanism is load-induced muscle fiber detachment. For instance, the most common form of CMD (MDC1A) is caused by mutations in the basement membrane component *laminin alpha 2* (*LAMA2*) [62] and in zebrafish with mutations in the *LAMA2* ortholog, muscle contractions lead to muscle fiber detachment and loss [63]. This mechanism is consistent with exercise-induced CK elevation in *Col4a1* mutant mice. This mechanism is also consistent with the observation that *C. elegans Col4a1* mutants die with ruptured basement membranes shortly after muscle contractions begin [45], [55]. A similar mechanism in alveolar basement membranes could also explain why newborn *Col4a1* mutant mice have respiratory distress immediately after starting to breathe [22].

Thus, myopathy resulting from mutations in basement membrane components *LAMA2* and *COL4A1* might share a common pathogenic mechanism whereby contraction-induced load leads to muscle fiber detachment. Given this potential shared mechanism and the phenotypic variability of *COL4A1* mutations, we propose that MDC1A patients that are negative for *LAMA2* mutations are suitable candidates for screening for mutations in *COL4A1* and *COL4A2*. Importantly, if a component of the pathology is secondary to deleterious consequences of compromised blood brain barrier and load-induced myopathy, there is the potential for therapeutic interventions to blunt the severity of this devastating disease. For example, conditions that promoted protein folding and increased COL4A1 secretion in *C. elegans* mutants were able to rescue muscle contraction-induced basement membrane disruptions and promoted viability and survival [55]. Thus, chemical chaperones, or other methods to promote protein folding, might have therapeutic potential in human patients and improve the prognosis for MEB/WWS patients.

## Materials and Methods

### Ethics statement

#### Animals

Experiments were conducted in compliance with protocols approved by the Institutional Animal Care and Use Committee at University of California in San Francisco.

#### Human patients

Written, informed consent was obtained from the subjects or their legal guardians. The Institutional Review Boards of Beth Israel Deaconess Medical Center and Children's Hospital Boston and the UCSF Committee on Human Research approved this study.

### Retinal analyses

Heads were equilibrated in 20% sucrose in phosphate buffered saline (PBS) overnight at 4°C, embedded in OCT compound (Sakura Finetek), and flash frozen using dry ice/ethanol. For each genotype we analyzed six eyes at E14 and E16 and eighteen eyes at E18. For each eye, at least 3 position-matched, transverse sections (20 µm) were co-labelled with antibodies against Islet-1 (1∶10, Developmental Studies Hybridoma Bank 39.4D5sup and laminin (1∶1000, Abcam), or Islet-1 and activated Caspase-3 (1∶1000, R&D Systems). Sections were blocked (Tris-buffered saline with 0.1% Triton-X (TBST) containing 5% normal goat serum (Invitrogen)) for 1 hour at room temperature (RT) and washed twice for 2 min with TBS. Labeling was performed in TBS containing 3% BSA (Sigma) overnight at 4°C. Sections were incubated with Alexa Fluor 488 goat anti-mouse and Alexa Fluor 594 goat anti-rabbit secondary antibodies (1∶1000, Invitrogen) for 2 hours at RT and cover-slipped with Vectashield Hard Set with DAPI (Vector Labs).

### Cerebral analyses

Adult brains were fixed by trans-cardiac perfusion with 4% paraformaldehyde (PFA) in 0.1 M sodium phosphate, pH 7.4 then post-fixed in PFA at 4°C overnight and prepared for cryosectioning as described above. For Nissl staining, coronal sections (25 µm) were hydrated for 5 min in buffer (0.2% sodium acetate, 0.3% glacial acetic acid) then incubated for 10 min in Nissl stain (0.02% cresyl violet in buffer). Sections were washed in water (2×2 min) and de-stained for 15 sec (0.3% glacial acetic acid in 70% ethanol) before being dehydrated, cleared in xylene and cover-slipped with Permount (Fisher).

For GFAP and NeuN immunostaining, sections were incubated in 1% H202 in PBS for 15 min, washed in PBS, blocked in PBS containing 5% NGS, 0.1% Triton-X, and 1% BSA in PBS for 1 hour at RT. Sections were incubated with anti-GFAP antibody (1∶500, Chemicon) or anti-NeuN antibody (Chemicon 1∶500) overnight at 4°C, washed in PBS containing 0.1%Triton-X, and incubated for 2 hr with biotinylated goat anti-rabbit or goat anti-mouse antibody (1∶500, Vector Labs). Sections were then incubated in avidin-biotin solution (Vector Labs) for 90 min and immunoreactivity was visualized by treating sections with diaminobenzidine (DAB, Vector Labs). Sections were dehydrated, cleared in xylene and cover-slipped with Permount (Fisher).

For BrdU labeling, pregnant mice were injected with BrdU (50 mg/kg) at gestational days 14, or 16. At P0, heads were fixed in 4% PFA for 4 hr, embedded in OCT, snap frozen in dry ice/ethanol bath. Cryosections (25 µm) were incubated in sodium citrate buffer (10 mM sodium citrate, pH 6.0, 0.05% Tween-20) for 30 min starting at 90°C and allowed to cool, rinsed in water, incubated in 1 M HCl containing 0.2 mg/ml pepsin for 10 min at RT, in 2 N HCl for 20 min at 37°C. Sections were then washed and labeled with anti-BrdU (1∶30, Accurate Chemical) and biotinylated goat anti-rat secondary (1∶500, Vector Labs). For laminin immunolabeling of P0 brains, heads were processed as described in the retinal analyses section and 20 µm coronal sections were immunolabeled with anti- laminin antibody (1∶1000, Abcam).

### Muscle analyses

Peak grip force was determined using a Grip Strength Meter (Columbus Instruments) and using the average from 3 consecutive trials on each animal. For CK measurements, blood was drawn before and after exercise from the tail vein in a hematocrit tube, centrifuged for 5 min, and the serum was collected. CK activity was measured using CK-NADP assay (Raichem) and a microplate reader (Biorad). Exercise was 30 minutes with a 15° downhill grade on a treadmill equipped with a shock plate (Columbus Instruments). Animals were started at 7 m/min and increased 3 m/min every 2 min until maximum of 16 m/min speed was reached. Non-peripheral nuclei were evaluated in quadriceps and tibialis anterior muscles dissected and frozen in liquid nitrogen-cooled isopentane. Cryosections (8 µm) were stained with hematoxylin and eosin for histopathology or labeled with anti-pan laminin antibody (Sigma), followed by an anti-rabbit AlexaFluor-488 secondary antibody, and the nuclei were labeled with DAPI. The observers were masked to genotypes and counted between 2000–5000 fibers per animal.

For dystroglycan enrichment and immunoblotting, quadriceps muscle biopsy (100 mg) was homogenized in TBS (pH 7.5) containing 1% Triton-X and protease inhibitors (Thermo Scientific Pierce). The soluble fraction was incubated overnight at 4°C with 200 ul of wheat germ agglutinin (WGA)-agarose beads (Vector Laboratories) to enrich for glycosylated proteins. Beads were washed three times with 1 ml TBS containing 0.1%Triton-X and protease inhibitors (Thermo Fisher) and were boiled in SDS-PAGE loading buffer for 5 minutes. Proteins (50 µl) were separated on 10% SDS-PAGE and transferred to polyvinylidene fluoride (PVDF) membranes (BioRad). Membranes were blocked for 2 hours at room temperature in 5% non-fat milk in TBS containing 0.1% Tween-20, incubated overnight with a mouse monoclonal anti- α-dystroglycan antibody recognizing the glycosylated form of α-dystroglycan (IIH6; 1∶1000, Millipore), or rabbit polyclonal anti- β-dystroglycan antibody (raised against the C-terminus of dystroglycan precursor; 1∶400, Santa Cruz) diluted in blocking buffer. Membranes were washed in TBS containing 0.1% Tween-20, incubated 2 hours at room temperature with horseradish peroxidase-conjugated secondary antibody raised in donkey (anti-mouse IgM, and anti-rabbit IgG, respectively; 1∶10 000, Jackson Immunoresearch) diluted in blocking buffer. Immunoreactivity was visualized using chemiluminescence (SuperSignal West Pico Chemiluminescent Substrate, Thermo Scientific).

For immunostaining, muscle cryosections (25 µm) were incubated with either β-dystroglycan (1∶150) or with α-dystroglycan (1∶400) antibody diluted in blocking buffer (10% normal donkey serum, 0.2% BSA in PBS containing 0.1% Triton-X) overnight at 4°C and incubated with Alexa Fluor 488 conjugated donkey anti-rabbit (Invitrogen) or cy5- conjugated donkey anti-mouse IgM (Jackson Immunoresearch).

For COL4A1 immunolabeling, muscle cryosections (25 µm) were fixed in acetone for 10 min, rinsed in TT buffer (50 mM Tric-HCl, pH 7.4, 0.1% Tween-20), incubated in acid solution (0.1 M KCl/HCl, pH 1.5), washed three times in TT buffer and incubated for an hour in blocking buffer (10% normal donkey serum and 2 mg/ml BSA in Tris buffer). Sections were then incubated with rat anti- COL4A1 (H11) monoclonal antibody (1∶200, Shigei Medical Research Institute, Japan) diluted in TT buffer overnight at 4°C and for 1 hour with Alexa Fluor 488 conjugated donkey anti-rat secondary antibody (Invitrogen) and cover-slipped using mowiol mounting medium (Calbiochem).

### Microscopy

Images were captured using an AxioImager M1 microscope equipped with an AxioCam MRm digital camera for fluorescence or AxioCam ICc3 for brightfield and AxioVision software (Zeiss).

### Human analyses

Genomic DNA (10 ng/µL) sequencing was performed using ABI BigDye v3.1 and analyzed using Sequencher software (Gene Codes Corporation). Control samples are from ethnically matched adults with no history of neurological disease.

### Functional analysis of human mutations

HT1080 Human fibrosarcoma cells were transfected using Superfect reagent (Qiagen) with the expression vector pReceiver–M02 vector (GeneCopoeia) containing a CMV promoter upstream of GFP (to evaluate transfection efficiency), wild-type *Col4a1* or mutant *Col4a1* cDNA cloned and a neomycin resistance cassette, allowing stably transfected cells to be selected using G418 (Invitrogen). After 12 days of G418 selection (600 mg/ml), individual surviving clones were isolated and expanded in presence of 600 mg/ml of G148.

Stably transfected HT1080 cells were cultured in Dulbecco's modified Eagle's medium (DMEM) supplemented with penicillin, streptomycin, nonessential amino acids, glutamine, sodium pyruvate, G418 (250 mg/ml for maintenance), 10% Fetal Bovine Serum (FBS) and ascorbic acid (50 mg/ml) at 37°C in 5% CO2 in a humid atmosphere until they reach 80–90% confluence. Then, cells were serum-deprived for 24 hours under the same culture conditions and harvested and protein were extracted from HT1080 cells using cell extraction buffer containing 0.05 M Tris-HCl pH 8.0, 0.15 M NaCl, 5.0 mM EDTA, 1% NP-40, and protease inhibitors (Pierce) at 4°C. After centrifugation (13000 rpm, 20 min at 4°C), the soluble fraction of the whole cell lysate was collected for subsequent Western blot analysis. The conditioned medium was collected at the same time and supplemented with protease inhibitors.

Determination of protein concentration in the soluble fraction was performed using a DC protein assay (BioRad) and 45 ug of total protein were separated on a 4–15% gradient SDS-PAGE under non-reducing conditions and transferred to polyvinylidene fluoride (PVDF) membranes (BioRad). For the conditioned medium, the volume loaded on the gel was adjusted based on the protein concentration of the soluble fraction, which is assumed to reflect the number of cells for a given sample. Membranes were blocked for 2 hours at room temperature in 5% non-fat milk in TBS containing 0.1% Tween-20, and overnight at 4°C in 3% BSA in TBS. Membranes were then incubated with rat anti- COL4A1 (H11) monoclonal antibody (1∶100, Shigei Medical Research Institute, Japan) in 1% BSA in TBS for 3 hours at room temperature and were washed in TBS containing 0.1% Tween-20, incubated 2 hours at room temperature with horseradish peroxidase-conjugated secondary antibody raised in donkey (anti-Rat IgG 1∶10 000, Jackson Immunoresearch) diluted in 5% non-fat milk in TBS containing 0.1% Tween-20. Immunoreactivity was visualized using chemiluminescence (SuperSignal West Pico Chemiluminescent Substrate, Thermo Scientific). Densitometric analysis was performed on low exposure images using the NIH Image J software (National Institutes of Health). For quantitative analysis of the ratio of secreted to intracellular mutant COL4A1 protein, the amount of COL4A1 detected in the conditioned medium was divided by the amount of intracellular COL4A1 normalized with actin.

### Statistical analyses

In experiments where two groups were compared, samples were compared by a Student's T-test with p<0.05 considered significant. In experiments where more than two groups were compared, samples were analyzed by one-way ANOVA followed by a Tukey's post hoc test with p<0.05 considered significant. For calculation of apoptotic index, one E18 *Col4a1^+/Δex40^* eye had an extremely high apoptotic index (12.9) that was considered an outlier by Grubbs' test (p<0.01 vs. all mutants and Z = 2.93248; vs. all samples Z = 3.26997) and was removed from subsequent statistical analyses.

## Supporting Information

Figure S1 *Col4a1^+/Δex40^* mice have hippocampal neuronal localization defects. Representative cresyl violet stained coronal sections from adult *Col4a1^+/+^* (A–D) and position-matched *Col4a1^+/Δex40^* (E–H) brains revealed subtle but consistent neuronal localization defects in hippocampi of mutant mice. Regions of the CA1 (red boxes in A and E), CA3 (black boxes in A and E) and dentate gyrus (white boxes in A and E) are enlarged in B and F, C and G, and D and H, respectively. All mutant mice (n = 6) had hippocampal defects that included focal distortions (red box in E, compared to A) as well as diffusion of pyramidal cell layers in the CA1 (F, compared to B) and CA3 (G, compared to C) regions and granular cell layers in the dentate gyrus (H, compared to D). Scale bars: A and E, 500 µm; B–D and F–H, 100 µm.
(TIF)

Figure S2 Presence of reactive astrocytes in the brain of *Col4a1^+/Δex40^* mice. Representative coronal brain sections from adult *Col4a1^+/+^* (A–C) and position matched *Col4a1^+/Δex40^* (D–F) mice labeled with anti-GFAP antibody revealed the presence of reactive astrocytes. Compared to *Col4a1^+/+^* brains (A), *Col4a1^+/Δex40^* mice had increased GFAP labeling in the cortex (D). Higher magnification images revealed increased labeling in *Col4a1^+/Δex40^* mice (E compared to B) and the astrocytic morphology of the labeled cells. Increased labeling was also detected in *Col4a1^+/Δex40^* hippocampi compared to *Col4a1^+/+^* (F compared to C). Scale bars: A, C, D, F 500 µm; B and E, 100 µm.
(TIF)

Figure S3 COL4A1 is present in skeletal muscle basement membrane of *Col4a1* ^+/+^ and *Col4a1* ^+/Δex40^ mice. Immunohistochemical labeling of muscle sections with antibodies against COL4A1 confirmed that COL4A1 is present in *Col4a1^+/+^* (A) and *Col4a1^+/Δex40^* (B) skeletal muscle basement membrane and revealed that the mutant basement membrane appeared to label less uniformly and less intensely.
(TIF)

Figure S4 Brain imaging of patients 1 and 2. Brain magnetic resonance imaging in patient 1 (A–B), a normal control (C–E) and patient 2 (F–G). Patient 1 has a typical WWS phenotype with a severe cobblestone-type cortical malformation with a smooth brain surface that resembles lissencephaly, a thin and discontinuous laminar heterotopia just below the cortex (arrow in A), diffusely abnormal white matter (asterisk in A), thin brainstem, enlarged tectum (arrowhead in B), moderate kink at the midbrain-pons junction (arrow in B), severe cerebellar hypoplasia (seen in both A and B), and enlarged posterior fossa. The cortex is ∼10 mm thick with an irregular gray-white border (A), while classic lissencephaly is typically 12–20 mm thick with a smooth gray-white border. The brainstem kink is less severe than typical for WWS. Patient 2 has a less severe MEB phenotype with diffuse cobblestone-type cortical malformation that appears thicker over the frontal lobes (best seen in G), diffuse abnormal white matter signal (asterisk in G), moderately enlarged lateral ventricles (F and G) and thin brainstem with flat pons (arrow in F). The gyral pattern is irregular and resembles polymicrogyria, but no actual microgyri are seen. These are patients LP93-014 and LP90-029 from the Dobyns database. Parts A and B are modified from Figure 1 in [Kanoff, et al]. [Kanoff RJ, Curless RG, Petito C, Falcone S, Siatkowski RM, et *al.*, (1998) Walker-Warburg syndrome: neurologic features and muscle membrane structure. Pediatr Neurol 18:76–80.].
(TIF)

Table S1 Sequencing primers for *COL4A1*.
(PDF)

Table S2 Non-Synonymous coding variants identified.
(PDF)

Table S3 Synonymous coding variants identified.
(PDF)

Table S4 Intronic variants identified.
(PDF)

Text S1 Clinical details of human patients.
(DOC)
