# Supplementary figures and images for: COL4A1 Mutations Cause Ocular Dysgenesis, Neuronal Localization Defects, and Myopathy in Mice and Walker-Warburg Syndrome in Humans
Source: PLoS Genet. 2011 May 19;7(5):e1002062. doi: 10.1371/journal.pgen.1002062 (PMC3098190; doi:10.1371/journal.pgen.1002062)

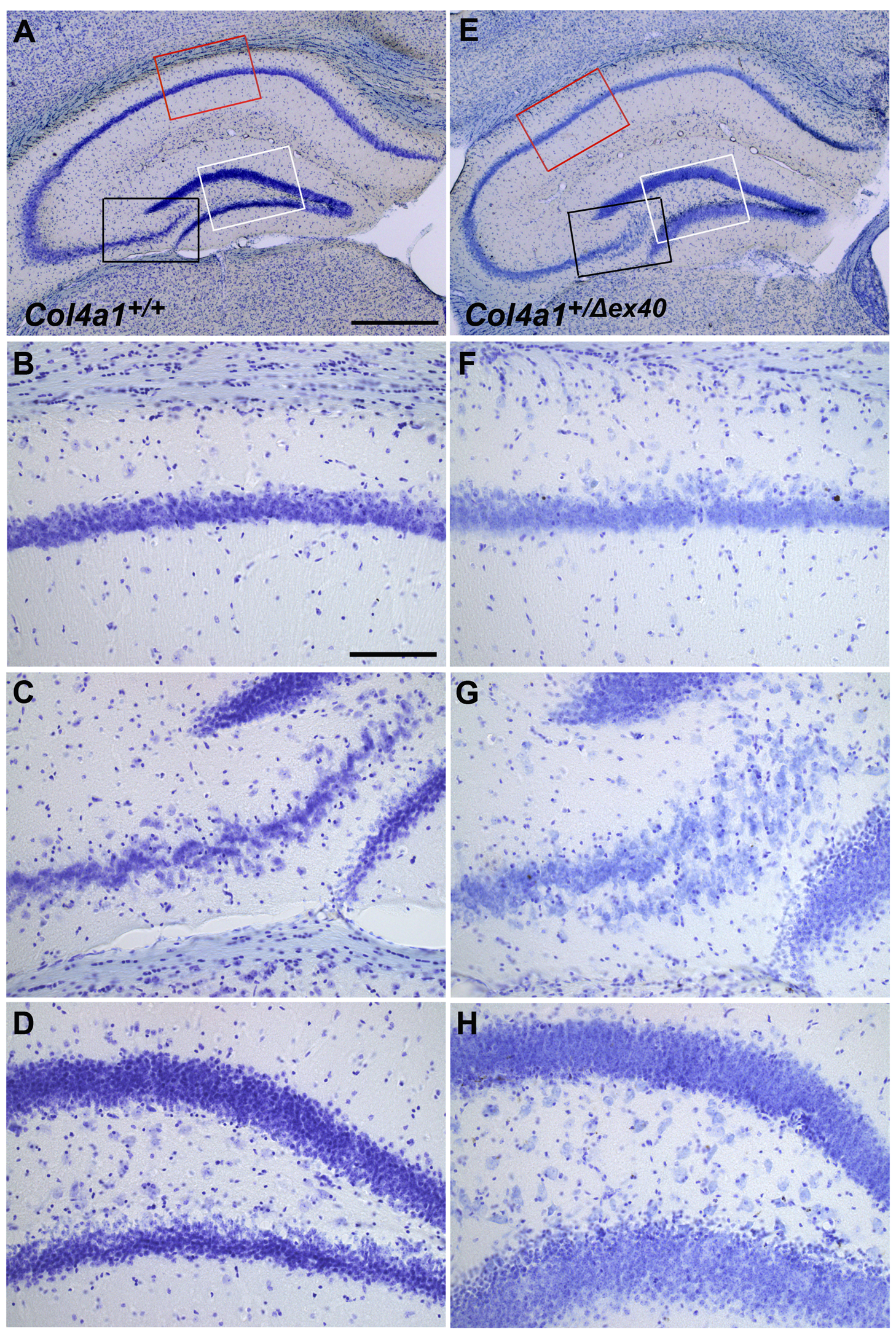

Supplement: Figure S1 — Col4a1+/Δex40 mice have hippocampal neuronal localization defects. Representative cresyl violet stained coronal sections from adult Col4a1+/+ (A–D) and position-matched Col4a1+/Δex40 (E–H) brains revealed subtle but consistent neuronal localization defects in hippocampi of mutant mice. Regions of the CA1 (red boxes in A and E), CA3 (black boxes in A and E) and dentate gyrus (white boxes in A and E) are enlarged in B and F, C and G, and D and H, respectively. All mutant mice (n = 6) had hippocampal defects that included focal distortions (red box in E, compared to A) as well as diffusion of pyramidal cell layers in the CA1 (F, compared to B) and CA3 (G, compared to C) regions and granular cell layers in the dentate gyrus (H, compared to D). Scale bars: A and E, 500 µm; B–D and F–H, 100 µm. (TIF) [file pgen.1002062.s001.tif]

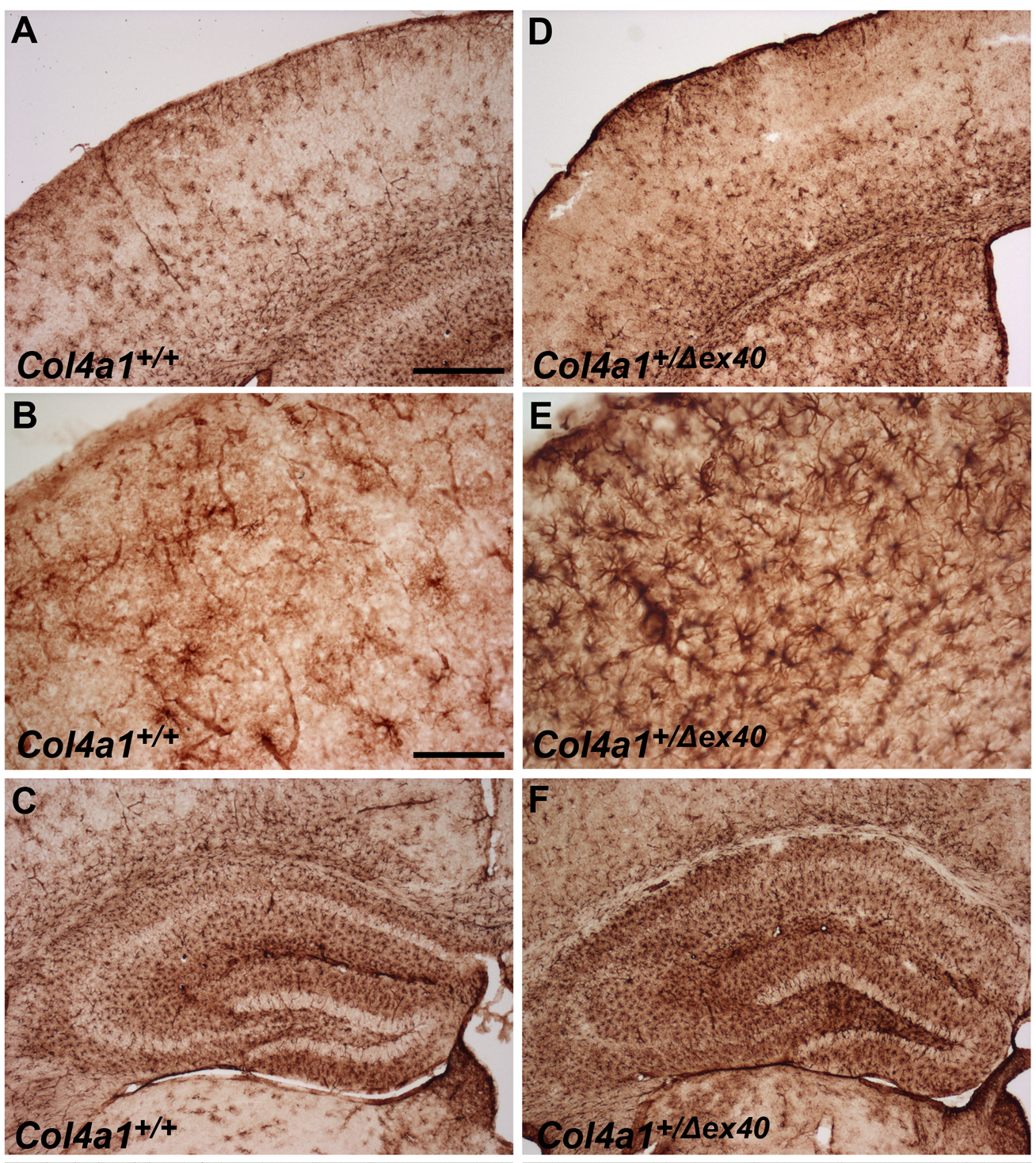

Supplement: Figure S2 — Presence of reactive astrocytes in the brain of Col4a1+/Δex40 mice. Representative coronal brain sections from adult Col4a1+/+ (A–C) and position matched Col4a1+/Δex40 (D–F) mice labeled with anti-GFAP antibody revealed the presence of reactive astrocytes. Compared to Col4a1+/+ brains (A), Col4a1+/Δex40 mice had increased GFAP labeling in the cortex (D). Higher magnification images revealed increased labeling in Col4a1+/Δex40 mice (E compared to B) and the astrocytic morphology of the labeled cells. Increased labeling was also detected in Col4a1+/Δex40 hippocampi compared to Col4a1+/+ (F compared to C). Scale bars: A, C, D, F 500 µm; B and E, 100 µm. (TIF) [file pgen.1002062.s002.tif]

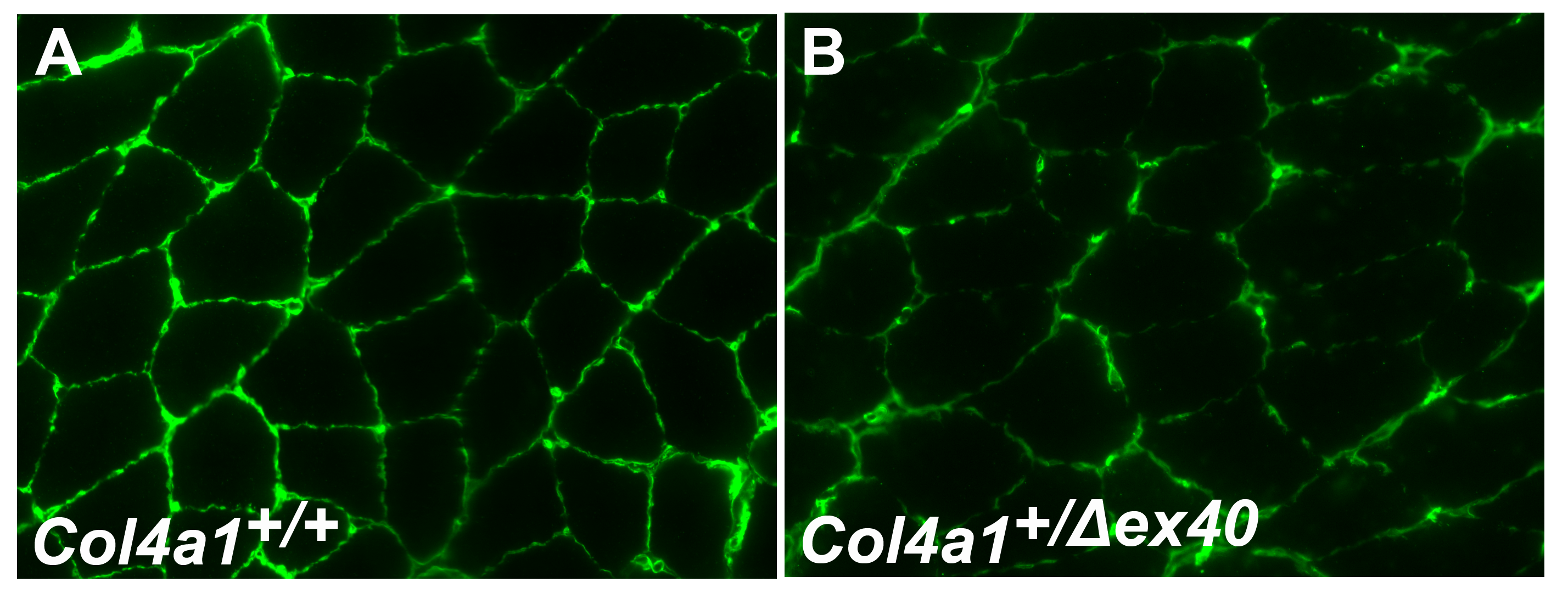

Supplement: Figure S3 — COL4A1 is present in skeletal muscle basement membrane of Col4a1 +/+ and Col4a1 +/Δex40 mice. Immunohistochemical labeling of muscle sections with antibodies against COL4A1 confirmed that COL4A1 is present in Col4a1+/+ (A) and Col4a1+/Δex40 (B) skeletal muscle basement membrane and revealed that the mutant basement membrane appeared to label less uniformly and less intensely. (TIF) [file pgen.1002062.s003.tif]

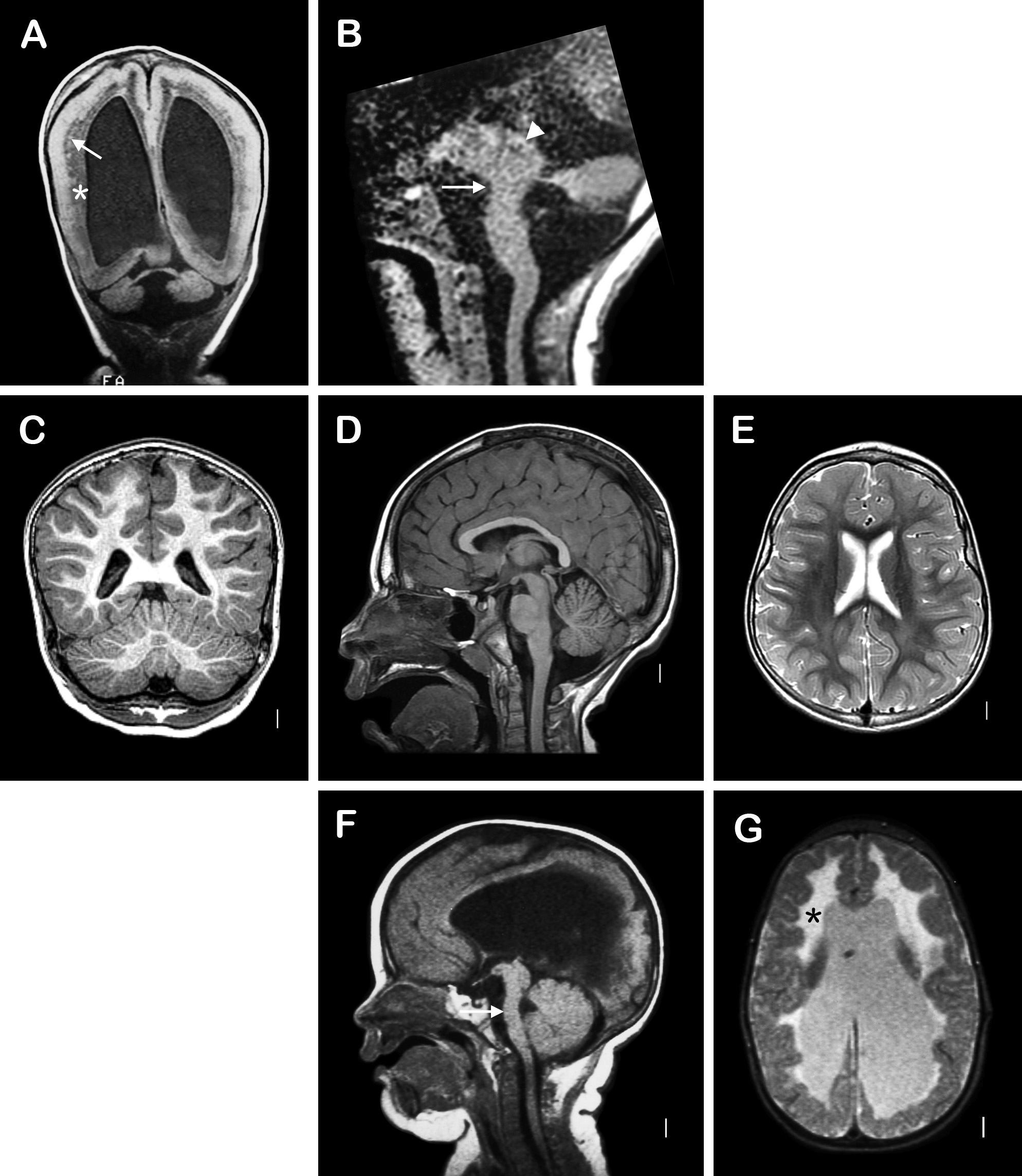

Supplement: Figure S4 — Brain imaging of patients 1 and 2. Brain magnetic resonance imaging in patient 1 (A–B), a normal control (C–E) and patient 2 (F–G). Patient 1 has a typical WWS phenotype with a severe cobblestone-type cortical malformation with a smooth brain surface that resembles lissencephaly, a thin and discontinuous laminar heterotopia just below the cortex (arrow in A), diffusely abnormal white matter (asterisk in A), thin brainstem, enlarged tectum (arrowhead in B), moderate kink at the midbrain-pons junction (arrow in B), severe cerebellar hypoplasia (seen in both A and B), and enlarged posterior fossa. The cortex is ∼10 mm thick with an irregular gray-white border (A), while classic lissencephaly is typically 12–20 mm thick with a smooth gray-white border. The brainstem kink is less severe than typical for WWS. Patient 2 has a less severe MEB phenotype with diffuse cobblestone-type cortical malformation that appears thicker over the frontal lobes (best seen in G), diffuse abnormal white matter signal (asterisk in G), moderately enlarged lateral ventricles (F and G) and thin brainstem with flat pons (arrow in F). The gyral pattern is irregular and resembles polymicrogyria, but no actual microgyri are seen. These are patients LP93-014 and LP90-029 from the Dobyns database. Parts A and B are modified from Figure 1 in [Kanoff, et al]. [Kanoff RJ, Curless RG, Petito C, Falcone S, Siatkowski RM, et al., (1998) Walker-Warburg syndrome: neurologic features and muscle membrane structure. Pediatr Neurol 18:76–80.]. (TIF) [file pgen.1002062.s004.tif]
