# Supplementary material for: COL4A1 Mutations Cause Ocular Dysgenesis, Neuronal Localization Defects, and Myopathy in Mice and Walker-Warburg Syndrome in Humans
Source: PLoS Genet. 2011 May 19;7(5):e1002062. doi: 10.1371/journal.pgen.1002062 (PMC3098190; doi:10.1371/journal.pgen.1002062)
Supplement: Table S1 — Sequencing primers for COL4A1. (PDF) [file pgen.1002062.s005.pdf]

Table 1: Sequencing Primers

Primer Name	Primer Sequence	Primer Length
COL4A1_x_30_F	GCTTGAAAAGGGTTGAGCAG	20
COL4A1_x_30_R	GCCTCTAAGATTTGCATCGTTC	22
COL4A1_x_31_F	CCTAGAGCTCGTGGCTTTCC	20
COL4A1_x_31_R	CTTCACTTTATAGGGACCCCG	21
COL4A1-1F_3	ATTGGCCCTTCCAGAAGCA	19
COL4A1-1R_3	CCAGTACCCATACCATAACAAAGGAG	26
COL4A1_x11_F	CACCTGCTAGGAGTGGGGAG	20
COL4A1_x11_R	TTGACTAAGGGATGGATGAAAG	22
COL4A1_x12_F	GCCTGAAATCTACCATCTTATTGC	24
COL4A1_x12_R	AGTCCAGACATTGATCCAAAGG	22
COL4A1_x13_F	TTTAGGCTTTTACCTCTCTCTGC	23
COL4A1_x13_R	CTGAGTTTTGACCCATTTTCTC	22
COL4A1_x14-15_F	CAAGTGAGTGCTGGAGATCAAC	22
COL4A1_x14-15_R	AGAAGTCCCTACGAGCCTTTTC	22
COL4A1_x16_F	GAGAAGCAGTAACCGTCAGAGG	22
COL4A1_x16_R	CAGAGCTTTGGTGTTATATCCGTTA	25
COL4A1_x17_F	CACATAGGAACAAGGCGAACTC	22
COL4A1_x17_R	TGGTTTTTACAAGCAGGCTCAC	22
COL4A1_x18_F	GGCACACTCTATTTTACCAGAAG	23
COL4A1_x18_R	CAGTGCTCTCACAGACCCAG	20
COL4A1-19F_3	CCTGACCTTGTGATCCACCT	20
COL4A1-19R_3	AGACCTGGTCACAGACAGTCCT	22
COL4A1_x2_F	CATCTCCATTTGCATAGTGCC	21
COL4A1_x2_R	CACCTGAATTGCTGATTATTCG	22
COL4A1_x20_F	ACACTTACCAATGCACCAAGC	21
COL4A1_x20_R	CGTAAGATTGCTACCGATTGTG	22
COL4A1_x21_F	CTTTTCAGTGATGGTCTGGTTG	22
COL4A1_x21_R	CGCCTTTCTATTACACTCTGGC	22
COL4A1_x22_F	TGGTTTTATAATAGAGGTTGAGTTAGGAG	29
COL4A1_x22_R	GCTCCAAAGCCGGTAAGTATG	21
COL4A1_x23_F	AATATGTTAGTGACAGCCTTGCC	23
COL4A1_x23_R	GAAAATCAGGCCTTCTATGGAG	22
COL4A1_x24_F	TATCCAAGCAATCATCCACTTC	22
COL4A1_x24_R	AATGCAAAACGACACAACTCTG	22
COL4A1_x25_F	AGGTGGCTTCTTGATTCTATGG	22
COL4A1_x25_R	GCCATCATCAAGGAGATAGAAATAC	25
COL4A1_x26_F	CAGAAGAATGCGATAAGGACAG	22
COL4A1_x26_R	CCAATTCCTGGAAGAATCAAAG	22
COL4A1_x27-28_F	AGGGAAGAAGTGGAGAACACAG	22
COL4A1_x27-28_R	CAGCTTCTGTGGTGTTTTGATG	22
COL4A1_x29_F	CCTCCACAGTTGACCTTCTCAG	22
COL4A1_x29_R	GCTACGTTCTCTGATACTTTCTATTCTATG	30
COL4A1_x32-33_F	TCTGCAGAAAGTCATAGTTAAACAG	25
COL4A1_x32-33_R	TGATCTATGACAACTTGAGAATTTCC	26
COL4A1_x3-4_F	TATAGCCATCACAGTTGGTCAC	22
COL4A1_x3-4_R	AGGAGAAAGGAGGGAAAAGG	20
COL4A1_x34-35_F	ACAAGAACGTGAGCCTTTTGAG	22
COL4A1_x34-35_R	TCTACAGAAAGAGCTGGGGAAC	22
COL4A1_x36_F	AGCCAGGTACAGTGTGGAGC	20
COL4A1_x36_R	CCTGGATTCTGTCCGTCTCAG	21
COL4A1_x37_F	CTTGTATCTCATTCTTGGCTGG	22
COL4A1_x37_R	GACTCCCTGTGTGTTATGGCTC	22
COL4A1-38F2	CCTCTACctgcttctcctttga	22
COL4A1-38R2	GAAACCAGATGGTTGGCTCTAC	22
COL4A1-39F_3	TGCAGGGAGATCAAGGGATA	20
COL4A1-39R_3	TCCTGCTAGTGACAAGACCTCA	22
COL4A1_x40_F	CAAAGGGCCATTTTGTATGG	20
COL4A1_x40_R	TTATTCACTGAACACAGGCAGTC	23
COL4A1_x41_F	TAGTGCTTTGTGTTGTACCTGC	22
COL4A1_x41_R	GAAGACACTGCCCCTTGTTC	20
COL4A1_x42_F	GTTTCCTGTGGTGTCCCTGG	20
COL4A1_x42_R	GCCAAACACAATTTCAAGCTG	21
COL4A1_x43_F	TCTGTCTCAAGGGTTGTCACTG	22
COL4A1_x43_R	AAGAAAGAACTGATTTAGTGGGG	23
COL4A1_x44_F	TTCCAACTAGCAGGTTACTGGAG	23
COL4A1_x44_R	GGTGCTATCAGTGCTAAGGTGC	22
COL4A1_x45_F	GCAGGTGTATGAATGTGTATATATGG	26
COL4A1_x45_R	TTGCTGCAAAGAATTCCCTATC	22
COL4A1_x46_F	TTCTCCTTCTAAAGTTGTTTCCG	23
COL4A1_x46_R	AGATACATGGGTGAGGAGGAAC	22
COL4A1_x47_F	GCTCCTTGAACACTGAAAAGG	21
COL4A1_x47_R	ATGCCTGGTTCTGCTCCTTC	20
COL4A1_x48_F	CATGAATAGGTAGGATACTTTGTGG	25
COL4A1_x48_R	AGTGTTTCACTCGCTACTGCC	21
COL4A1_x49_F	TGTCCAGCTGAAATCCACTTTAC	23
COL4A1_x49_R	AATGGATTCAACGTTTTGGAG	21
COL4A1_x50_F	ATGTTGAGTGTAGGTAACGGGG	22
COL4A1_x50_R	CGAGATGCAGAGAACTCCAAG	21
COL4A1_x51_F	AGTGTGTGGCTTTCCAGAGG	20
COL4A1_x51_R	CATCTTTGAGACTTTGCATTGG	22
COL4A1_x52_F	GGCTGATCCCTTAGAGATTTCC	22
COL4A1_x52_R	ATTGGACAGTGAGGTACACAGG	22
COL4A1_x5-6_F	ATGAGGGCATGACTCCATTAAC	22
COL4A1_x5-6_R	TTTCCAATTGTGGCAAATAAAAC	23
COL4A1_x7-8_F	AGTGATGCATGCGATGTCAG	20
COL4A1_x7-8_R	GAATGTGCTTCTATGGCACTTG	22
COL4A1_x9-10_F	TGCCAACTATCAAAGAGTTTTCAG	24
COL4A1_x9-10_R	GAGCGACCACAACTGTGTAAAG	22
