# Supplementary material for: COL4A1 Mutations Cause Ocular Dysgenesis, Neuronal Localization Defects, and Myopathy in Mice and Walker-Warburg Syndrome in Humans
Source: PLoS Genet. 2011 May 19;7(5):e1002062. doi: 10.1371/journal.pgen.1002062 (PMC3098190; doi:10.1371/journal.pgen.1002062)
Supplement: Table S2 — Non-Synonymous coding variants identified. (PDF) [file pgen.1002062.s006.pdf]

Table 2: Non-Synonymous Coding Variants

Amino Acid	Codon	Position	Genotype (number of patients)		
Val 7 Leu	**G**TC --> **C**TC	within signal peptide	G/G (10)	C/G (11)	C/C (5)
Met 1016 Val	**A**TG --> **G**TG	Y position of repeat	A/A (26)	A/G (1)	G/G (0)
Gln 1316 Glu	**C**AA --> **G**AA	Y position of repeat	C/C (26)	C/G (1)	G/G (0)
Gln 1334 His	CA**A** --> CA**C**	Y position of repeat	A/A (11)	A/C (13)	G/G (3)
