# Supplementary material for: COL4A1 Mutations Cause Ocular Dysgenesis, Neuronal Localization Defects, and Myopathy in Mice and Walker-Warburg Syndrome in Humans
Source: PLoS Genet. 2011 May 19;7(5):e1002062. doi: 10.1371/journal.pgen.1002062 (PMC3098190; doi:10.1371/journal.pgen.1002062)
Supplement: Table S3 — Synonymous coding variants identified. (PDF) [file pgen.1002062.s007.pdf]

Table 3: Synonymous Coding Variants

Amino Acid	Codon	Genotype (number of patients)		
Ala 144 Ala	GC**A** --> GC**T**	A/A (10)	A/T (10)	T/T (7)
Pro 419 Pro	CC**T** --> CC**C**	T/T (16)	T/C (9)	C/C (2)
Pro 605 Pro	CC**T** --> CC**C**	T/T (26)	T/C (1)	C/C (0)
Pro 710 Pro	CC**G** --> CC**A**	G/G (14)	G/A (13)	A/A (0)
Glu 733 Glu	GA**G** --> GA**A**	G/G (26)	G/A (1)	A/A (0)
Gly 1061 Gly	GG**G** --> GG**A**	G/G (13)	G/A (14)	A/A (0)
Arg 1063 Arg	CG**A** --> CG**T**	A/A (13)	A/T (14)	T/T (0)
Leu 1324 Leu	CT**C** --> CT**T**	C/C (26)	C/T (1)	T/T (0)
Gly 1332 Gly	GG**C** --> GG**T**	C/C (26)	C/T (1)	T/T (0)
Ala 1490 Ala	GC**C** --> GC**T**	C/C (17)	C/T (7)	T/T (3)
Ala 1599 Ala	GC**G** --> GC**A**	G/G (26)	G/A (1)	A/A (0)
Ser 1600 Ser	TC**C** --> TC**T**	C/C (17)	C/T (8)	T/T (2)
