# Supplementary material for: COL4A1 Mutations Cause Ocular Dysgenesis, Neuronal Localization Defects, and Myopathy in Mice and Walker-Warburg Syndrome in Humans
Source: PLoS Genet. 2011 May 19;7(5):e1002062. doi: 10.1371/journal.pgen.1002062 (PMC3098190; doi:10.1371/journal.pgen.1002062)
Supplement: Table S4 — Intronic variants identified. (PDF) [file pgen.1002062.s008.pdf]

Table 4: Intronic Variants

Intronic Position		Genotype (number of patients)			
-6	from exon 1	G/G (12)	G/T (11)	T/T (2)	G/A (1)
-11	from exon 2	T/T (18)	T/C (7)	C/C (2)	
+8	from exon 3	C/C (23)	C/T (1)	T/T (3)	
+64	from exon 4	G/G (22)	G/A (2)	A/A (3)	
+15	from exon 8		delATTTT (2)		
+24	from exon 10	C/C (18)	C/T (7)	T/T (2)	
+59	from exon 10	T/T (14)	T/G (11)	G/G (2)	
-11	from exon 11	C/C (21)	C/G (5)	G/G (1)	
+88	from exon 11	C/C (15)	C/T (10)	T/T (2)	
+68	from exon 11	AC/AC (15)	AC/GT (10)	GT/GT (2)	
+7	from exon 13	A/A (14)	A/G (11)	G/G (2)	
-7	from exon 15	C/C (15)	C/G (11)	G/G (1)	
-10	from exon 16	C/C (13)	C/T (12)	T/T (2)	
+18	from exon 16	A/A (12)	A/G (13)	G/G (2)	
-36	from exon 21	C/C (6)	C/G (16)	G/G (5)	
+44	from exon 21	G/G (24)	G/A (3)	A/A (0)	
+58	from exon 23	T/T (25)	T/C (2)	C/C (0)	
+29	from exon 26	A/A (13)	A/G (14)	G/G (0)	
+86	from exon 26	C/C (7)	C/T (18)	T/T (2)	
-16	from exon 28	G/G (23)	G/A (4)	A/A (0)	
+75	from exon 29	G/G (14)	G/A (13)	A/A (0)	
-69	from exon 30	C/C (23)	C/T (4)	T/T (0)	
+51	from exon 35	C/C (12)	C/T (12)	T/T (1)	
-62	from exon 36	C/C (13)	C/T (13)	T/T (1)	
-31	from exon 36	A/A (13)	A/G (13)	G/G (1)	
+102	from exon 43	A/A (26)	A/G (1)	G/G (0)	
-9	from exon 44	T/T (11)	T/C (14)	C/C(2)	
+45	from exon 44	T/T (11)	T/C (14)	C/C(2)	
+48	from exon 44	C/C (11)	C/T (13)	T/T (3)	
-78	from exon 45	A/A (26)	A/G (1)	G/G (0)	
+38	from exon 46	C/C (10)	C/G (12)	G/G (5)	
+7	from exon 49	C/C (20)	C/T (7)	T/T (0)	
+8	from exon 49	G/G (26)	G/A (1)	A/A (0)	
+16	from exon 49	G/G (20)	G/A (7)	A/A (0)	
-32	from exon 50	G/G (25)	G/A (2)	A/A (0)	
