# Supplementary material for: COL4A1 Mutations Cause Ocular Dysgenesis, Neuronal Localization Defects, and Myopathy in Mice and Walker-Warburg Syndrome in Humans
Source: PLoS Genet. 2011 May 19;7(5):e1002062. doi: 10.1371/journal.pgen.1002062 (PMC3098190; doi:10.1371/journal.pgen.1002062)
Supplement: Text S1 — Clinical details of human patients. (DOC) [file pgen.1002062.s009.doc]

**SUPPORTING ONLINE MATERIAL**

**Clinical Details of Human Patients**

Patient 1 was diagnosed with Walker-Warburg Syndrome (WWS) and has been described in detail previously (patient 1 in ^1^). Briefly, he was a male born at term to 3rd cousin Hispanic parents and had severe eye and brain malformations typical of WWS, as well as congenital muscular dystrophy with neonatal serum creatine kinase 2,927 U/L, with no family history of similar conditions. At birth the child had macrocephaly, hypotonia and areflexia. The eyes were small and the right optic nerve was atrophic/dysplastic. CK was greater than 1000 (U/L) and muscle biopsy showed non-specific myopathic changes suggesting a dystrophic process: widespread atrophic process, hypertrophic myofibers and scattered myofibers in varying stages of necrosis and regeneration. Immunostaining for dystrophin was normal. Computed tomography and magnetic resonance imaging showed hydrocephalus, lissencephaly and Dandy-Walker malformation. Neonatal magnetic resonance imaging (MRI) demonstrated abnormalities pathognomonic for WWS including diffuse agyria with ~1 cm thick cortex, beaded subcortical heterotopia, severely enlarged lateral ventricles, and mildly short corpus callosum. Posterior fossa images demonstrated a dysplastic brainstem with enlarged and dysplastic tectum, severe kink at the midbrain-pons junction, small and flat pons, small and dysplastic cerebellum with the vermis more severe than the hemispheres, upward rotation of the vermis associated with cystic dilatation of the 4th ventricle, and probably enlarged posterior fossa. The cerebellar anomaly meets criteria for Dandy-Walker malformation but with the addition of marked foliar dysplasia. The baby was blind and did not reach any developmental milestones. A gastrostomy tube was used for feeding. He died at 6 months of age from severe respiratory impairment. This patient is listed as subject LP93-014 in the Lisdb developmental disorders database maintained by Dr. Dobyns, and as subject M1001M in the Brain and Tissue Banks for Developmental Disorders <http://www.pathology.med.miami.edu/btb/index.html>.

Patient 2 was born via vaginal delivery after a 42 ½ week gestation from a trigravida 33-year-old mother. The pregnancy was complicated by a diagnosis of gestational diabetes in the 3rd month. Parents were unrelated and the mother had one normal previous pregnancy and a spontaneous abortion. Birth weight was 3.96 kg and Apgar scores were 8 at 1 minute and 9 at 5 minutes. Occipital frontal circumference was large (36.5cm, 75th percentile), tone was somewhat low with normal reflexes. A Computed tomography scan was performed after a few post-natal weeks because of rapidly increasing head circumference and revealed hydrocephalus, which was shunted. At the age of 3 months he was also diagnosed with a seizure disorder, which was managed with Phenobarbital. Poor vision was noted and an ophthalmologic exam showed optic nerve hypoplasia, macular hypoplasia, narrowed retinal vessels and markedly reduced cone and rod responses. Hypotonia became more severe over time and he developed contractures in the distal limbs. At 13 months of age, he had no head control and was not able to sit up. CK was greater than 1000 (U/L), but no muscle biopsy was performed. He was severely delayed and functioned at the level of a 1-2 month-old. MRI imaging showed pronounced hydrocephalus, mild gyral abnormalities particularly frontally with normal cortical thickness, severe thinning of the corpus callosum and pontine hypoplasia. The cerebellum appeared normal. The diagnosis was in the spectrum between Walker-Warbug Syndrome and Muscle-Eye-Brain disease. Age of death is not known.

**REFERENCE**

f

1. Kanoff, R.J. et al. Walker-Warburg syndrome: neurologic features and muscle membrane structure. *Pediatric Neurology* **18**, 76-80 (1998).
